# Study on Preparation and Hydration Mechanism of Sand Washing Residue Mud-Based LC3 Cement by Mechanical–Thermal Activation

**DOI:** 10.3390/ma19173802

**Published:** 2026-09-07

**Authors:** Gang Wang, Keliang Li, Linhua Jiang, Junjie Ma, Yichuan Yan, Hengjun Hou, Huanqiang Liu, Weizhun Jin

**Affiliations:** 1 China Construction Seventh Engineering Division Co., Ltd., Zhengzhou 450004, China; wanggang--1988@163.com (G.W.); mjj634773267@163.com (J.M.); 15639316869@163.com (Y.Y.); houhengjunecit@163.com (H.H.); 2 School of Civil Engineering and Transportation, North China University of Water Resources and Electric Power, Zhengzhou 450045, China; klli73@163.com; 3 College of Civil and Transportation Engineering, Hohai University, Nanjing 210024, China; hhulhjiang@163.com; 4 College of Civil Engineering, Henan University of Engineering, Zhengzhou 451191, China

**Keywords:** sand washing residue mud, composite activation strategy, LC3 cement, proportion optimization design, hydration mechanism

## Abstract

To enhance the application value of sand washing residue mud (SWRM) and mitigate its adverse environmental impacts, this study focuses on the resource utilization of SWRM. The research employed a combined mechanical–thermal activation method to enhance the activity of SWRM and utilized the mixed optimal design module to design and optimize the mixing ratio of SWRM-based LC3 cement. The results showed that within a ball-milling time range of 3–9 min, as the ball-milling time increased, the specific surface area increased, the median particle size D_50_ decreased, and the particle size was mainly concentrated within the range of 0.1–30 μm; the specific surface area of the washed sand residue after 3 min of grinding reached 1080 m^2^/kg, with D_50_ being 3.50 μm, which met the requirements for making cementitious materials. After thermal activation at temperatures ranging from 450 °C to 950 °C for the 3 min ground SWRM, the 28 d activity index showed a trend of increasing first and then decreasing with the increase in calcination temperature, and the 28 d activity index reached the maximum of 79.3% at a calcination temperature of 650 °C. The optimal mixing ratio of the AC70 group’s SWRM-based LC3 cement obtained through the mixing design was: cement clinker 66.5%, desulfurized gypsum 3.5%, activated SWRM 15%, and limestone powder (LP) 15%. The 28 d compressive strength of the AC70 group’s SWRM-based LC3 cement was 34.1 MPa, with initial setting and final setting times of 170 min and 240 min respectively, and the volume stability was qualified. The microscopic test results indicated that under the synergistic effect of alkali and salt, the silicate and aluminosilicate tetrahedral structures in the active sand-washed mud (ASWRM) decomposed, forming a C-(A)-S-H network structure, which was the main source of strength for the SWRM-based LC3 cement in the later stage. The ecological benefit calculation and analysis showed that compared with ordinary Portland cement of the same grade, the AC70 group’s SWRM-based LC3 cement had a 27.6% reduction in implicit energy consumption, a 44.1% reduction in carbon emissions, and a 25% reduction in cost. This study provides an innovative approach for the high value-added resource utilization of SWRM.

## 1. Introduction

Sand washing residue mud (SWRM) is a high-moisture solid waste generated during the water washing and screening process of aggregate production [1]. It is characterized by large output, extensive land occupation, and severe ecological pollution risks. The resource disposal of such waste has always been a research hotspot in the field of construction solid waste treatment. To date, domestic and foreign scholars have conducted extensive systematic research on the resource utilization of muddy by-products produced in mining and aggregate processing [2,3,4], which provides solid theoretical and technical support for the high-value recycling of SWRM.

Various activation techniques and proportioning strategies have been adopted to realize the resource utilization of muddy solid wastes in the construction industry. Yassine et al. [5] fabricated thermal-insulating building composites using gravel washing sludge as raw material, whose 21 d compressive strength reached 5.38 MPa, and compared with common thermal insulation materials, its thermal conductivity is slightly lower, while its specific heat capacity is higher. Vishojit et al. [6] activated washing sand sludge via sodium hydroxide treatment and calcination. The mortar prepared therefrom achieved a 56 d compressive strength of 1.86 MPa, and the results show that the residual sludge from sand washing can enhance its reactivity by being stimulated with alkali and activated through heat treatment; however, the intensity is relatively low, which limits its application. Febin et al. [7] manufactured concrete blocks with 50% manufactured sand sludge replacing fine aggregate, attaining a high 28 d compressive strength of 26.22 MPa. The SWRM can be used as a filling material to improve the microstructure of concrete, but its potential reactivity has not been utilized. Sanjuán, M.Á et al. [8,9] conducted a systematic study on ternary mixed cement composed of silica fume and limestone. The results showed that limestone not only filled the voids but also promoted the formation of C-S-H. Thus, it can be seen that the application of limestone as a cementitious material has a dual effect. Hu et al. [10] used manufactured sand washing sludge mud, lithium mica slag and cement as raw materials. When the proportion of manufactured sand washing sludge was 63%, the unfired and non-autoclaved bricks produced had a 28 d compressive strength of 15.3 MPa and a softening coefficient of 0.81. The SWRM has not yet fully utilized its potential hydration activity when used as aggregate. Based on the review of existing studies, the current resource utilization of sand washing residue mud still faces prominent problems, including low strength and low utilization rate, limited application scenarios and single activation methods. Most existing utilization approaches only realize low-value applications such as landfill [11], brick making and ceramsite preparation [12,13], which fail to achieve large-scale and high-value engineering promotion and restrict the industrial utilization of SWRM.

In order to enhance the application value and quantity of the SWRM, this study proposes a combined activation strategy integrating mechanical–thermal activation to prepare LC3 cement with activated sand washing residue mud (ASWRM) and limestone powder (LP) as mineral admixture. Mechanical grinding and high-temperature calcination were combined to significantly improve the pozzolanic activity of SWRM. The Mixture-Optimal Design module was adopted to optimize the mix proportion of SWRM-based LC3 cement. Furthermore, macroscopic working and mechanical performance tests combined with microscopic characterization were conducted to reveal the hydration mechanism and strength evolution law of the developed cement. This research provides a novel and high-value technical pathway for the resource utilization of SWRM and offers a reference for the industrial application of solid waste in low-carbon cement preparation.

## 2. Raw Materials and Experimental Methods

### 2.1. Raw Materials

**Figure 1:**
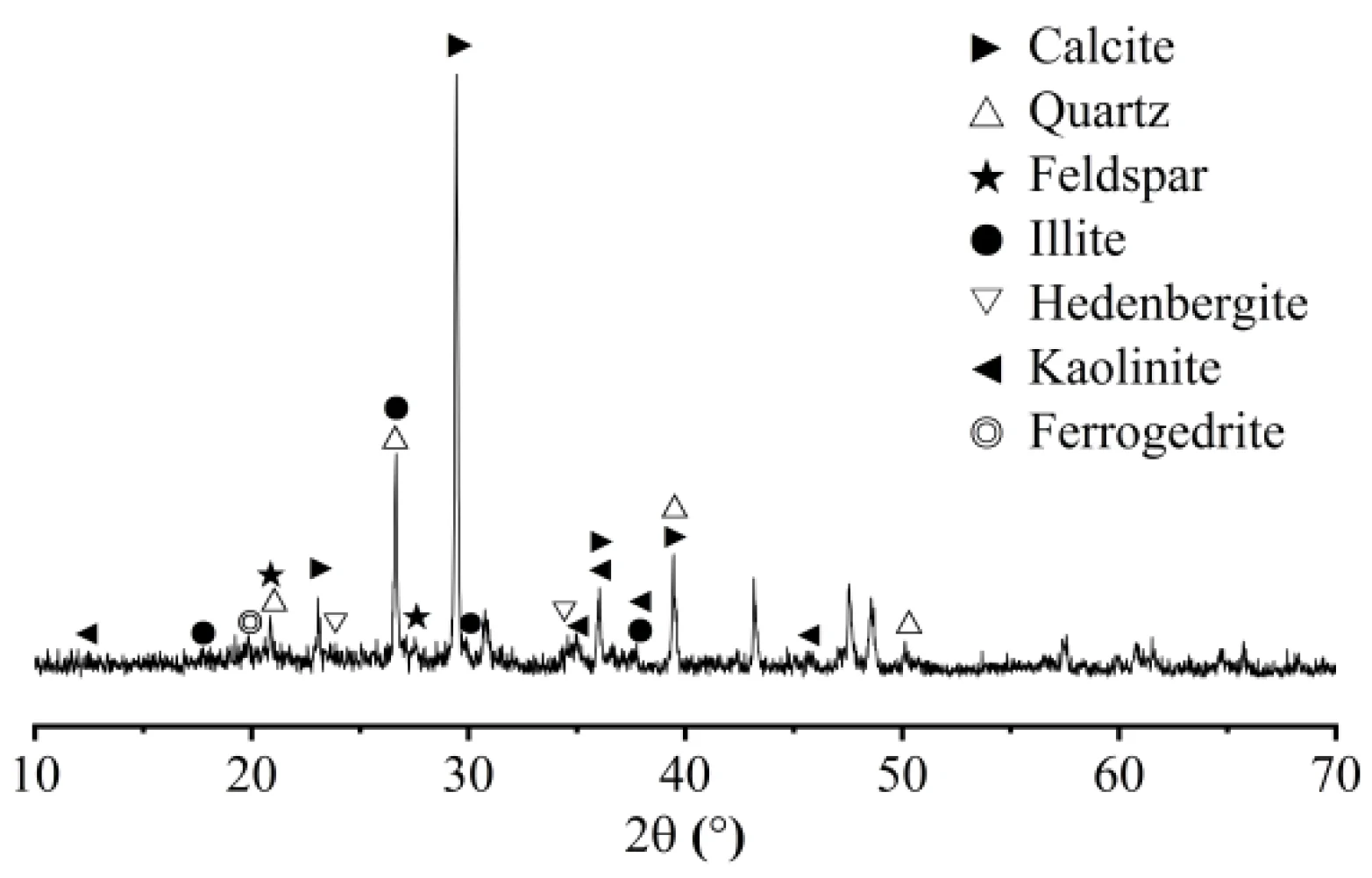
XRD patterns of SWRM.

**Figure 2:**
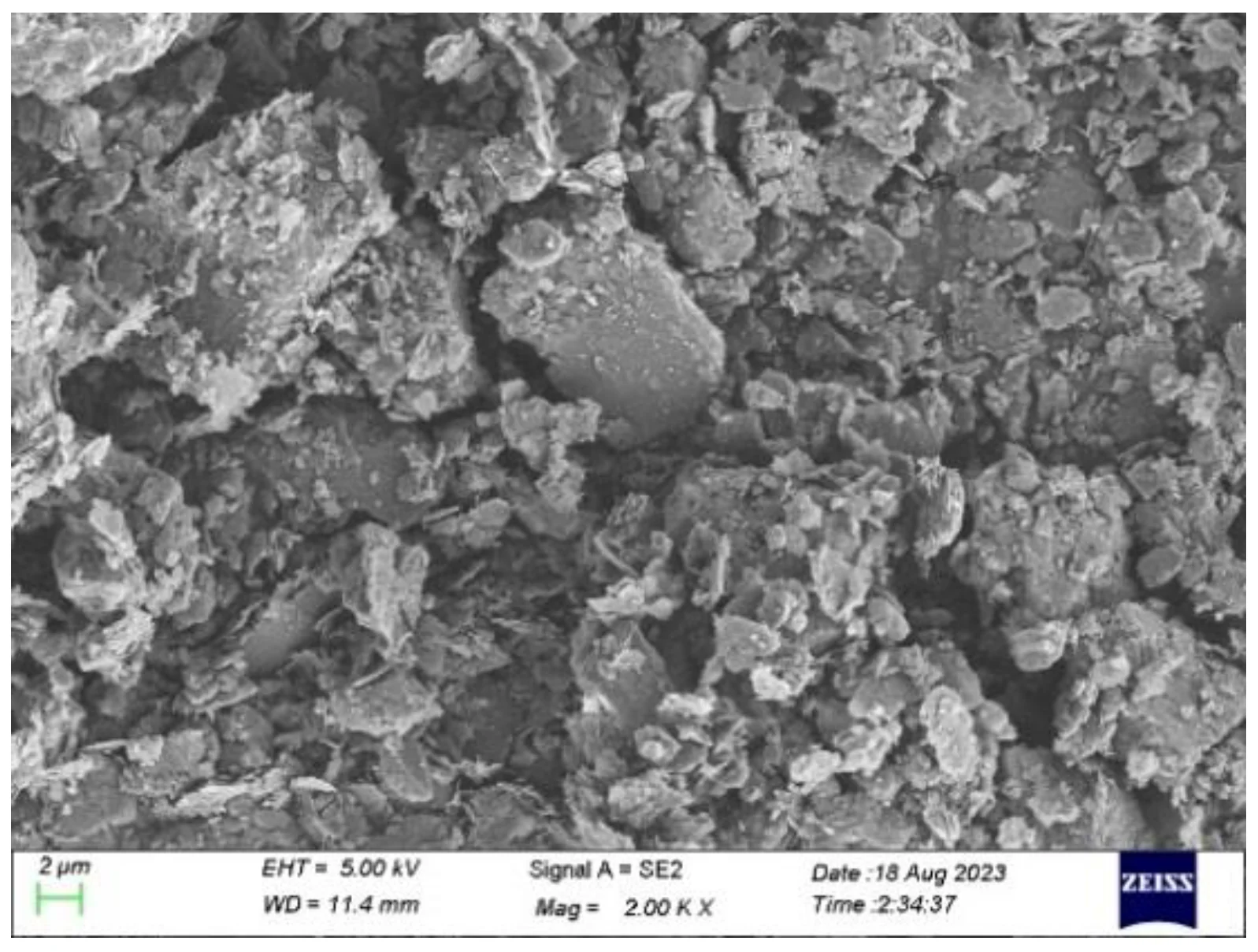
SEM image of SWRM.

SWRM was supplied by Henan Building Materials Research and Design Institute Co., Ltd. The raw sludge appears as pale yellow lumps. For the preparation of SWRM-based LC3 cement, the raw material was first dried at 105 ± 5 °C for 24 h and then ground into powder. The chemical compositions of the raw materials are listed in Table 1, and the XRD patterns and an SEM image of SWRM are shown in Figure 1 and Figure 2, respectively. It can be seen that the main mineral phases of SWRM are calcite and quartz, accompanied by a small amount of kaolinite and K-feldspar.

The cement clinker (CC) was ordinary Portland cement clinker obtained from a cement plant in Zhengzhou, and its chemical composition is presented in Table 1. The main mineral components are dicalcium silicate, tricalcium silicate, tricalcium aluminate, and tetracalcium ferroaluminate.

Limestone powder (LP) was purchased from Jinhui Environmental Protection Company in Lingshou County, Hebei Province, China. It is calcium-based LP, and its chemical composition is shown in Table 1. Its specific surface area reaches 1034 m^2^/kg, and the 28 d activity index is 69.8%.

Desulfurization gypsum (DG) was supplied by Guoneng Xingyang Thermal Power Plant in Zhengzhou, China. It is a by-product generated from wet flue gas desulfurization in power plants. The DG was dried at 45 °C for 24 h and ground in a ball mill for 35 min to obtain powder for testing. Its particle size D_50_ is 16.07 μm, the chemical composition is listed in Table 1, and the content of calcium sulfate dihydrate reaches 75%.

The P·O 42.5 ordinary Portland cement used in this experiment was commercially available. Its chemical composition is given in Table 1, and its performance parameters are summarized in Table 2.

The water reducer was white powdered polycarboxylate superplasticizer provided by Shandong Yousuo Chemical Technology Co., Ltd. (Linyi, China), with a water reduction rate no less than 18%.

### 2.2. Experimental Methods

**Figure 3:**
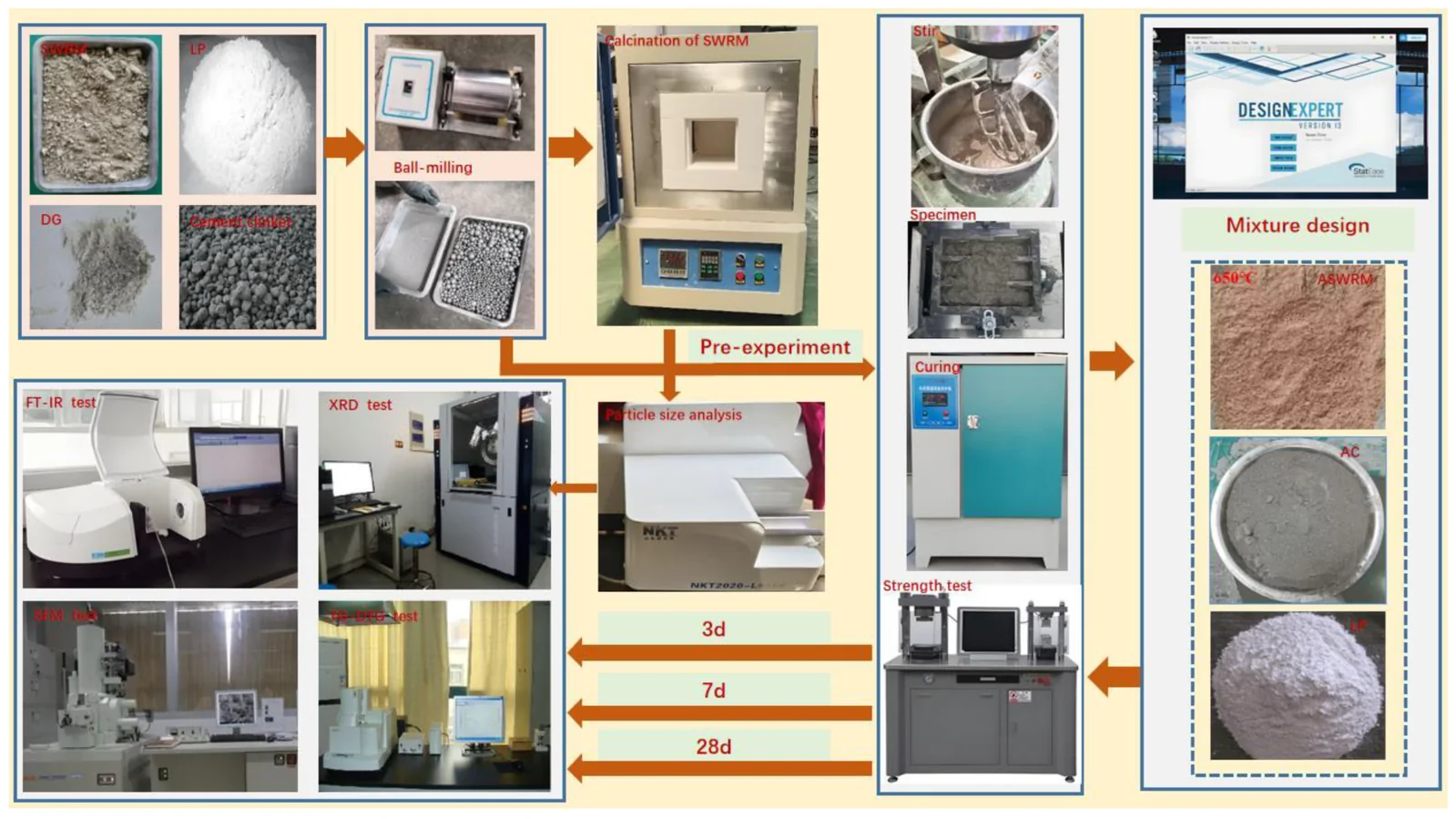
Sample preparation and testing methods.

Pure cement pastes with standard consistency were adopted in this test. The water requirement for standard consistency was determined in accordance with GB/T 1346—2024 [14]. Mortar tests were carried out following GB/T 17671—2021 [15]. The sample preparation and testing methods are shown in Figure 3.

All mortar specimens were cured under standard conditions at a temperature of 20 ± 2 °C and relative humidity above 95%. Mechanical tests were performed on a DYE-300 universal testing machine (Rongjida Instrument Technology Co., Ltd., Shanghai, China); the maximum range of the testing machine is 300 kN. During the test, the loading speed for the compressive strength was controlled at 2400 N/s ± 200 N/s, and the loading speed for the flexural strength was controlled at 50 N/s ± 10 N/s. The flexural and compressive strengths were tested at curing ages of 3 d, 7 d, and 28 d, and the final test results were averaged. The particle size distribution was measured by an NKT2020-L automatic laser particle size analyzer (Shandong Nanke Instrument Co., Ltd., Jinan, China).

Blocky or flaky fragments with a side length of approximately 1 cm were selected from the crushed specimens after strength tests for microstructural analysis. These samples were soaked in anhydrous ethanol for 24 h to stop the hydration reaction. Before the microscopic test, the samples needed to be dried. To minimize the influence of the drying temperature on the hydration products, the samples were dried in a vacuum drying oven at 60 °C for 24 h. Specimens for XRD and TG-DTG tests were further ground into powder with an agate mortar and sieved through a 0.15 mm sieve.

The XRD analysis was conducted on a D8 Advance X-ray diffractometer (Bruker AXS, Karlsruhe, Germany) with a Cu target. The operating voltage was 40 kV, and the current was 30 mA. The scanning range of 2θ was 10~70°, and the scanning rate was 8°/min.

A Regulus 8100 scanning electron microscope (SEM) (Hitachi High-Technologies Corporation, Tokyo, Japan) was used for microscopic morphology observation. The tested surface area of each sample was no less than 1 cm^2^. The samples were fixed with conductive adhesive and subjected to gold sputtering before observation.

Fourier transform infrared spectroscopy (FT-IR) was performed on a Nicolet-460 spectrometer (Thermo Fisher Scientific, Waltham, MA, USA) within the wavenumber range of 4000 cm^−1^ to 400 cm^−1^.

Differential thermogravimetry (DTG) was implemented on a DTG-60H analyzer (Shimadzu Corporation, Kyoto, Japan) with a nitrogen atmosphere. The temperature rose from 30 °C to 1000 °C at a heating rate of 10 °C/min.

## 3. Activation Treatment of SWRM

### 3.1. Mechanical Activation

**Figure 4:**
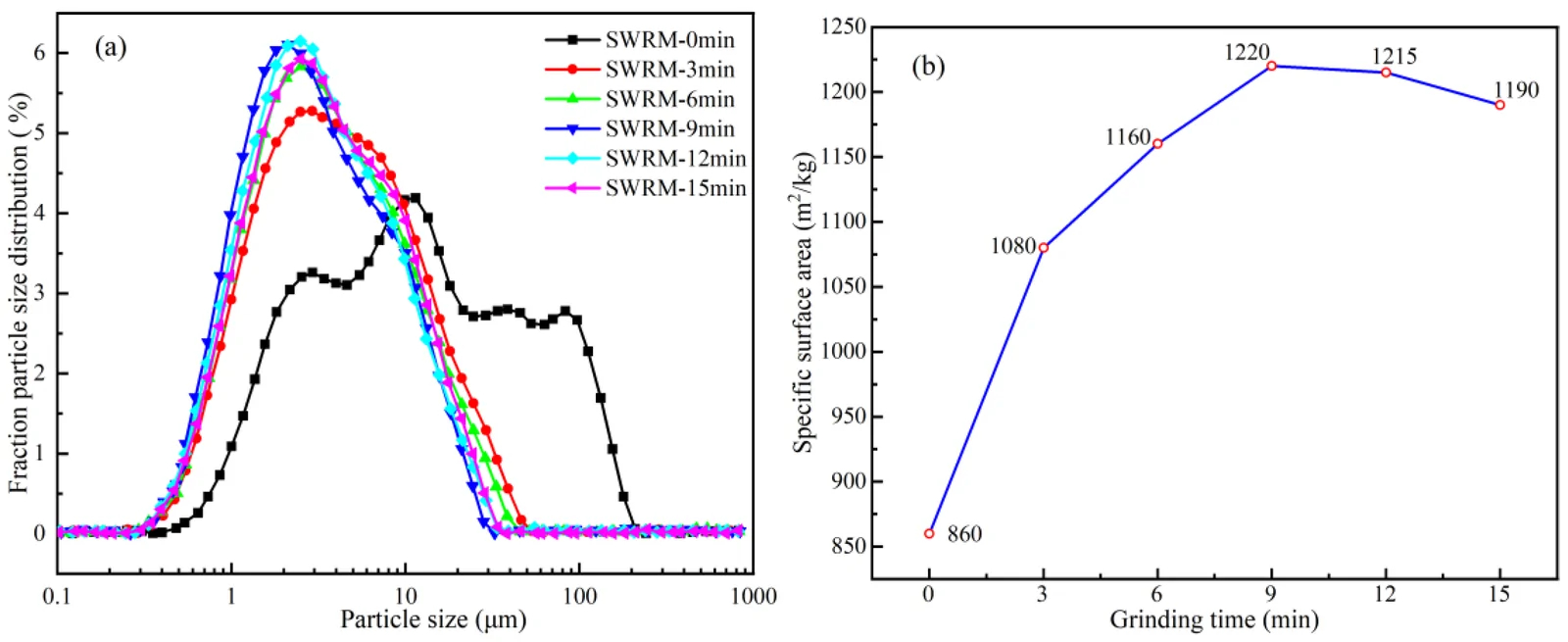
Effect of grinding time on particle size and specific surface area of SWRM: (**a**) differential particle size distribution; (**b**) specific surface area with different grinding durations.

Mechanical activation is a widely adopted modification technology for solid wastes [16]. To enhance the pozzolanic activity of SWRM, mechanical grinding was carried out as the first activation procedure in this study. Figure 4 presents the particle size distribution and specific surface area of crushed and dried raw mud subjected to different grinding durations.

As shown in Figure 4a, the differential particle size distribution of raw SWRM exhibits a multi-peak curve. The particle sizes are mainly concentrated in the range of 1–100 μm, and the proportion of particles larger than 100 μm is approximately 5%. With the extension of grinding time, the particle size changes remarkably. The fraction of particles larger than 30 μm gradually decreases, while the content of particles ranging from 0.2 μm to 5 μm increases continuously. When the grinding time increases from 0 min to 9 min, the distribution curve and its main peak shift leftward, accompanied by a reduction in the peak particle size. This phenomenon occurs because the coarse particles in the sludge are mostly agglomerates of fine grains. These agglomerates are easily broken and dispersed during ball milling, leading to fast particle refinement and high grinding efficiency. When the grinding time is prolonged from 9 min to 15 min, the distribution curve and main peak shift slightly to the right, and the corresponding peak particle size rises marginally. In the later stage of grinding, continuous collision and friction induce surface charge on fine particles, resulting in particle agglomeration under electrostatic force [17], which accounts for the slight increase in particle size.

Table 3 presents the test results of the characteristic particle sizes of washed sand sludge for different grinding times.

As shown in Table 3, with the increase in grinding time, the characteristic particle sizes D10, D50, and D90 of the washed sand residue all show a trend of first decreasing and then increasing. The change in particle size can be divided into two stages. At 9 min of grinding, the D_10_, D_50_, and D_90_ of the particles all reach their minimum values, with D_10_, D_50_, and D_90_ decreasing from the initial values of 1.66 μm, 9.89 μm, and 74.43 μm to 0.85 μm, 2.58 μm, and 10.17 μm respectively. During this stage, the large particles in the washed sand residue continue to be refined, and the particle size decreases rapidly. Continuing the grinding to 15 min, the D_10_, D_50_, and D_90_ of the washed sand residue particles show different degrees of growth, and the particle size distribution width decreases to the minimum value and then slightly increases. This is because the intermolecular forces between particles increase, resulting in agglomeration phenomena. Continued grinding would cause the particle size to increase, increase the energy consumption of the ball mill, and reduce the grinding efficiency.

It can be observed from Figure 4b that the specific surface area of SWRM first rises and then falls with increasing grinding time. The specific surface area of the raw mud is 860 m^2^/kg. After 3 min of grinding, the value reaches 1080 m^2^/kg, 1.26 times that of the raw material. The maximum specific surface area of 1220 m^2^/kg is achieved after 9 min of grinding, which is 1.4 times the initial value. Further grinding up to 15 min leads to a slight drop to 1190 m^2^/kg, which can be attributed to particle agglomeration. This trend is consistent with the variation in particle size distribution.

The grinding test demonstrates that after 3 min of ball milling, the particle sizes of SWRM are concentrated within 0.1–30 μm, and the specific surface area exceeds 1000 m^2^/kg. The D_10_, D_50_, and D_90_ of the particles are 0.98 μm, 3.50 μm, and 14.27 μm respectively. The D_50_ of common Portland cement is 10 μm. Considering the grinding efficiency and filling effect, the SWRM with 3 min of grinding meets the particle size requirements. Further grinding would not be economical. Therefore, the subsequent research used the SWRM after 3 min of grinding as the research object.

### 3.2. Thermal Activation

Thermal activation is another common method for solid waste modification. High-temperature calcination can alter the mineral compositions and crystal lattice structure of raw materials [18]. Considering the grinding efficiency and low-carbon requirements, SWRM ground for 3 min was thermally activated at temperatures of 450 °C, 550 °C, 650 °C, 750 °C, 850 °C, and 950 °C to determine the optimal calcination condition. Preliminary exploratory tests confirmed that the optimal calcination duration was 1 h.

**Figure 5:**
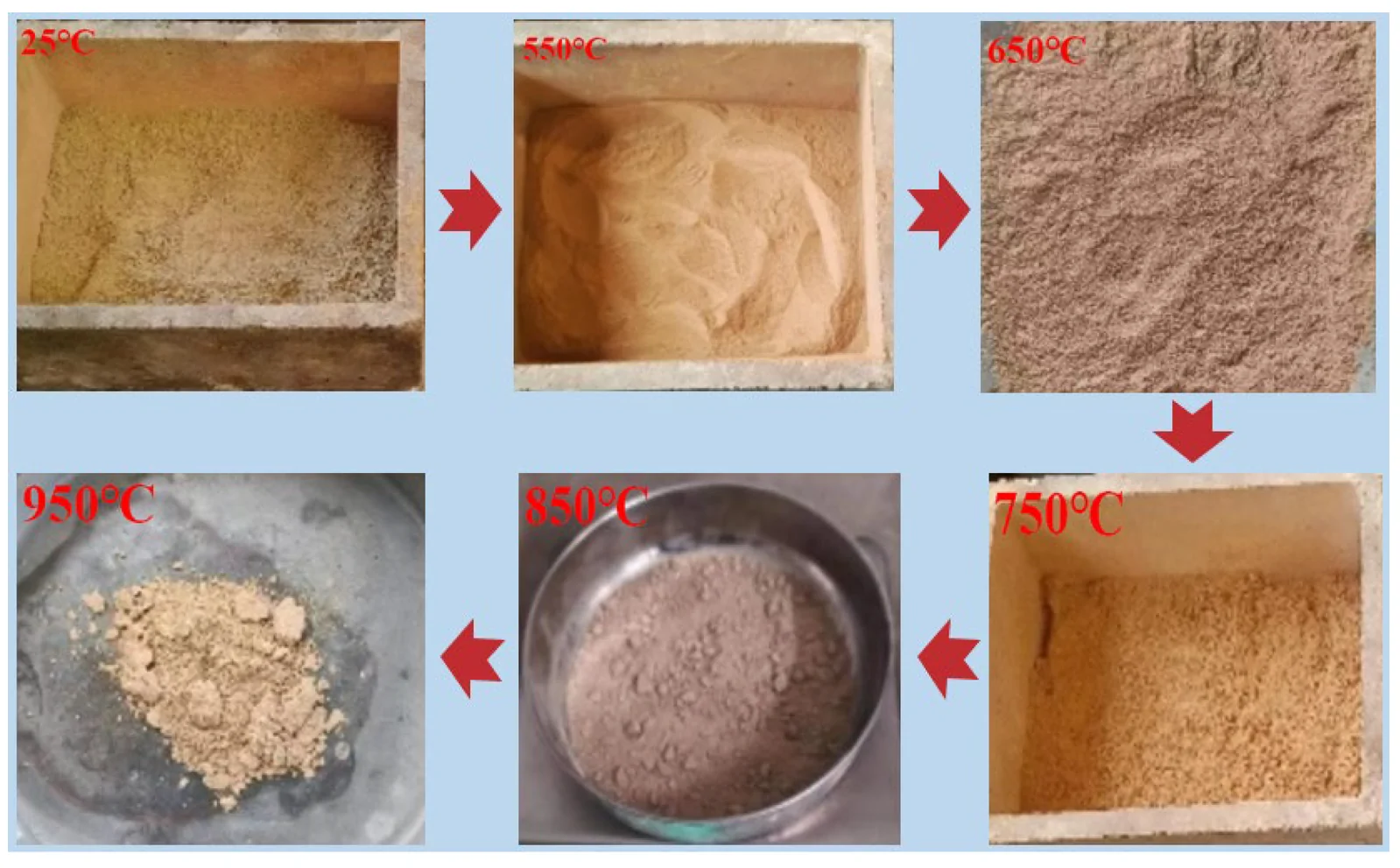
Change in color of SWRM after calcination at different temperatures.

Figure 5 shows the color variation of SWRM after calcination. It can be seen that the powder color changes gradually with the increase in calcination temperature, which indicates the transformation of mineral phases. When the calcination temperature rises from 550 °C to 750 °C, the color of the mud changes from yellow to light red, and the loose powder gradually becomes dense and coarse. Severe sintering occurs when the temperature reaches 850 °C and 950 °C.

**Figure 6:**
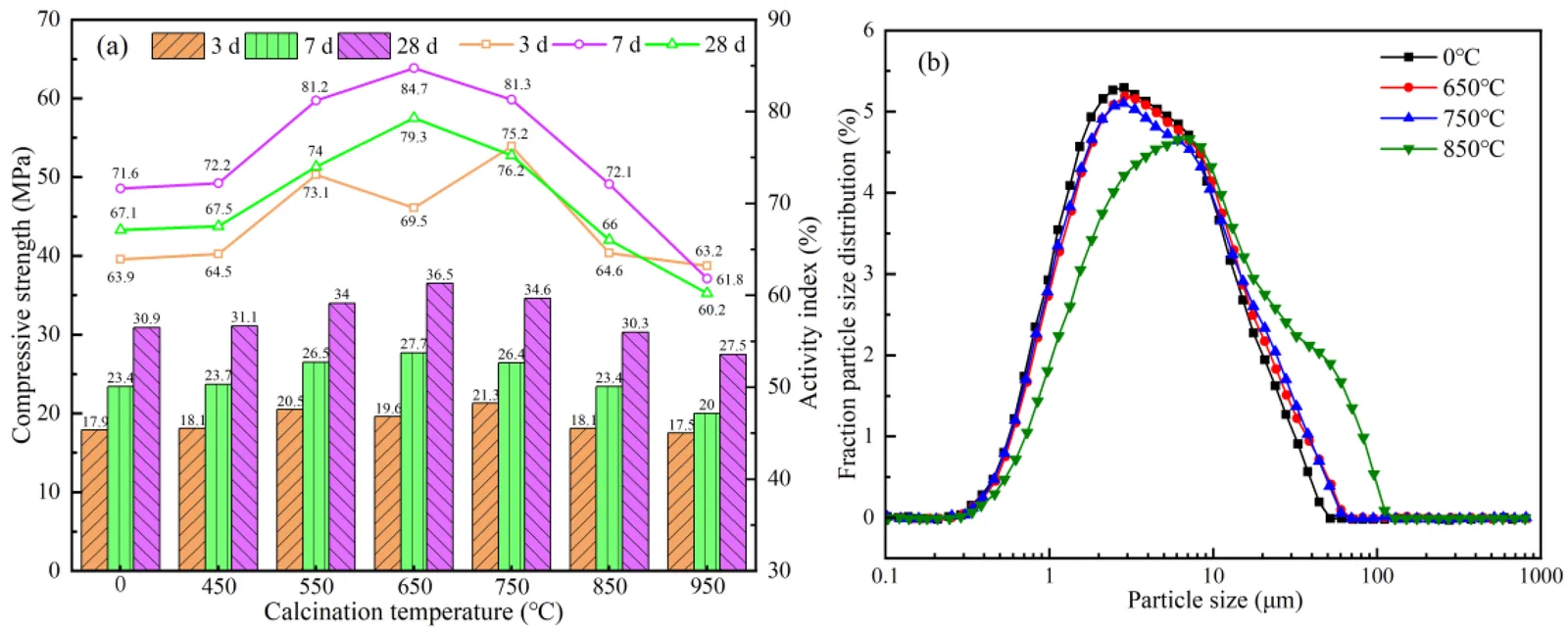
Effects of calcination temperature on compressive strength, activity index, and particle size of SWRM: (**a**) compressive strength and activity index; (**b**) differential particle size distribution.

To evaluate the effect of thermal activation, the activity index of calcined SWRM was tested. In this experiment, 30% of cement was replaced by calcined mud. The reference mix proportion was set as 450 g cement, 1350 g standard sand, with a water-to-cement ratio of 0.5. The 7 d and 28 d compressive strengths of the reference group were 32.6 MPa and 46.1 MPa, respectively. Figure 6 illustrates the compressive strength, activity index, and particle size distribution of specimens containing mud calcined at different temperatures.

As presented in Figure 6a, the compressive strength increases first and then decreases with rising calcination temperature, and the activity index follows the same trend. Compared with uncalcined mud, the 28 d activity index of samples calcined at 450 °C, 550 °C, 650 °C, 750 °C, and 850 °C increases by 0.7%, 5.9%, 12.3%, 8.2%, and −1.2%, respectively. The maximum 28 d activity index of 79.3% is obtained at the calcination temperature of 650 °C. When the temperature exceeds 850 °C, the activity index is slightly lower than that of raw mud. This is probably because amorphous alumina and silica gradually transform into inert mullite at high temperature [19,20].

Figure 6b shows the particle size distribution of partially calcined SWRM. Table 3 shows the results of particle size changes of SWRM at different calcination temperatures.

From Table 4 and Figure 6b, it can be seen that after the SWRM is calcined, the particle size increases. The D_50_ value increases from 3.5 μm to 5.72 μm. The cumulative distribution curve shifts slightly to the right, and the peak gradually moves downward. After the SWRM is calcined at 850 °C, the particle size changes significantly. The proportion of particles larger than 80 μm increases, and the specific surface area decreases with the increase in calcination temperature. This indicates that as the calcination temperature increases, the sintering phenomenon of the SWRM becomes more severe, which leads to a decrease in the activity of the SWRM.

After calcination at 650 °C, the 7 d and 28 d activity coefficients of the mud reach 84.7% and 79.3%. Among all groups, the mud ground for 3 min and then calcined at 650 °C achieves the optimal activation effect. This modified material was adopted to prepare LC3 cement in subsequent tests and is hereinafter referred to as activated sand washing residue mud (ASWRM).

**Figure 7:**
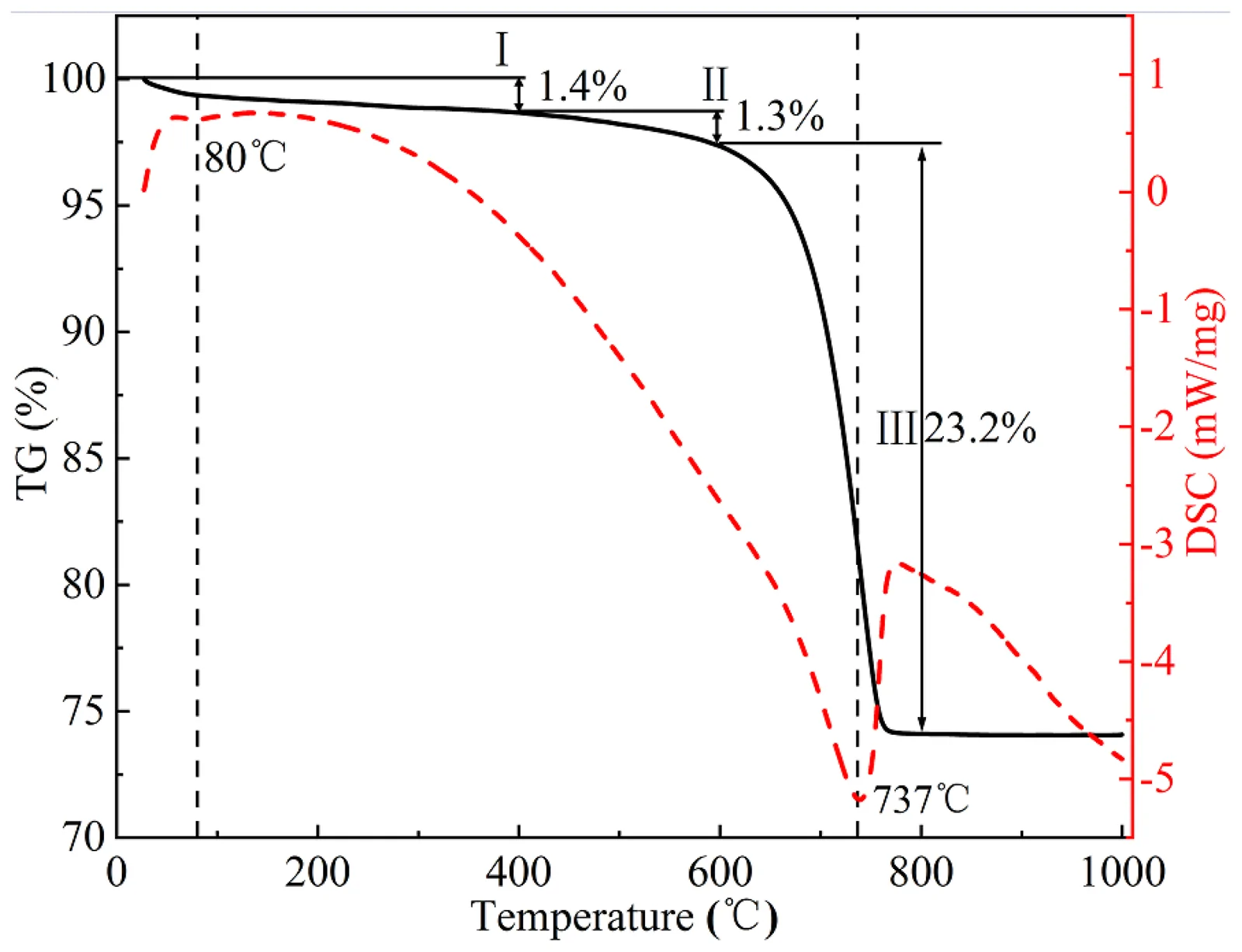
TG-DSC curves of SWRM.

The TG-DSC curve of the SWRM is shown in Figure 7. As shown in Figure 7, the process of heating the SWRM from room temperature to 1000 °C is a continuous weight loss process. The weight loss process in the TG curve can be divided into three stages. The first stage is from 23 °C to 400 °C, mainly caused by the evaporation of free water and adsorbed water in the SWRM and the combustion of organic substances [21]. The weight loss is 1.4%, and a weak endothermic peak appears at 80 °C in the DSC curve, which is due to the dehydration of the SWRM. The second stage is from 400 °C to 600 °C. During this stage, a significant decrease in mass can be observed. When the temperature exceeds 500 °C, kaolinite and illite begin to dehydroxylate, and the crystal structure is damaged, transforming into bentonite (Al_2_O_3_·2SiO_2_). The weight loss is 1.3%. The third stage is from 600 °C to 800 °C. During this stage, significant weight loss occurs, and the weight loss is the largest at 23.2%. The weight loss process almost ends at around 770 °C. The weight loss in this stage is mainly caused by the decomposition of carbonate minerals calcite under heating conditions, so the endothermic peak in the DSC curve is the strongest. At this time, the remaining hydroxyl groups of kaolinite and illite are completely removed, and amorphous aluminum oxide and silica with active properties are generated [22,23].

**Figure 8:**
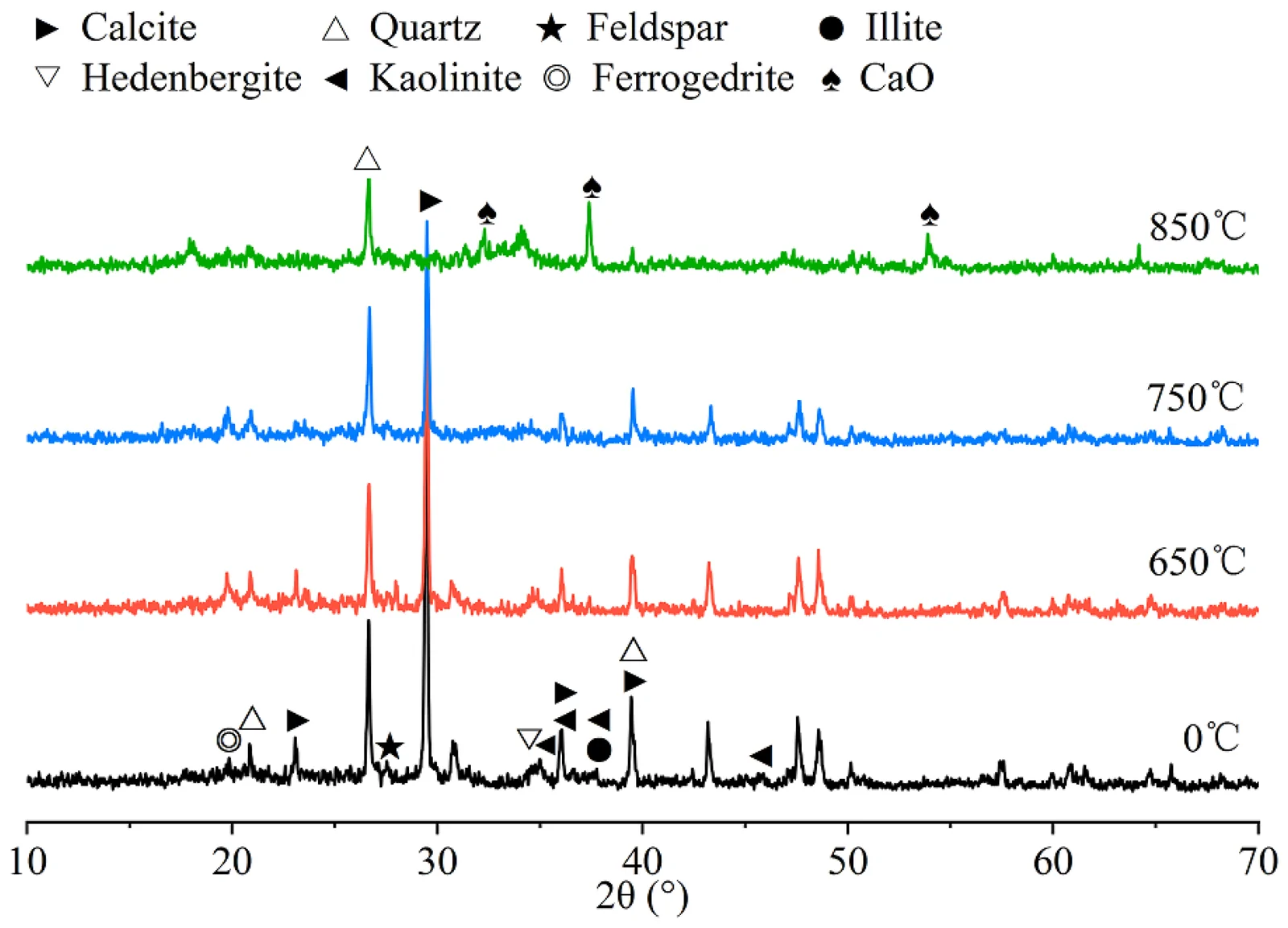
XRD patterns of SWRM at different calcination temperatures.

From the TG-DSC analysis in Figure 7, it can be seen that a lower calcination temperature makes some clay minerals and carbonates in the SWRM difficult to decompose, which is not conducive to the activation of the SWRM; an excessively high calcination temperature not only causes the SWRM to form lumps but also increase energy consumption and raise production costs. Therefore, a temperature of 650 °C, 750 °C, or 850 °C was selected for the thermal activation study. A shorter calcination time may lead to insufficient calcination of the SWRM, while a longer time increases energy consumption. Thus, a calcination time of 1 h was chosen. Figure 8 shows the XRD spectra of the SWRM at different calcination temperatures.

From Figure 8, it can be seen that compared with the original XRD spectrum, the characteristic peaks of the main mineral phases in the SWRM, such as quartz, calcite, kaolinite, potassium feldspar, and illite, undergo varying degrees of changes. After different temperatures of calcination, the characteristic peaks of quartz at 26.6° and 50.1° remain strong and do not disappear, indicating that the properties of quartz are relatively stable [24]. As the calcination temperature increases, the intensity of the characteristic peaks of calcite at 29.5° and 39.5° gradually weakens and disappears, and the characteristic peak of CaO appears at 850 °C. This is because calcite gradually decomposes and generates calcium oxide as the temperature rises. From Figure 7, it can also be seen that the characteristic peaks of kaolinite in the SWRM disappear at 650 °C, and no characteristic peak of Al_2_O_3_ is found. This indicates that the Al-O octahedron in kaolinite is destroyed, forming a tetrahedral aluminum atom, and kaolinite transforms into amorphous high-kaolinite and then generates amorphous Al_2_O_3_ and SiO_2_ [25]. From Figure 7, it can be seen that the silicate mineral phases such as illite, potassium feldspar, and calcium iron amphibole in the washed sand residue also decompose with the increase in temperature. The activity of the SWRM mainly comes from the increase in amorphous substances and the alkaline reaction provided by CaO reacting with water. The excessively high calcination temperature of the SWRM causes the formation of mullite from the amorphous substances, and the agglomeration and sintering of the SWRM particles become more serious, reducing the reaction contact surface, thereby resulting in a decrease in activity. Therefore, combined with the activity test results, a calcination temperature of 650 °C was selected for thermal activation, which can greatly stimulate the activity of the SWRM.

**Figure 9:**
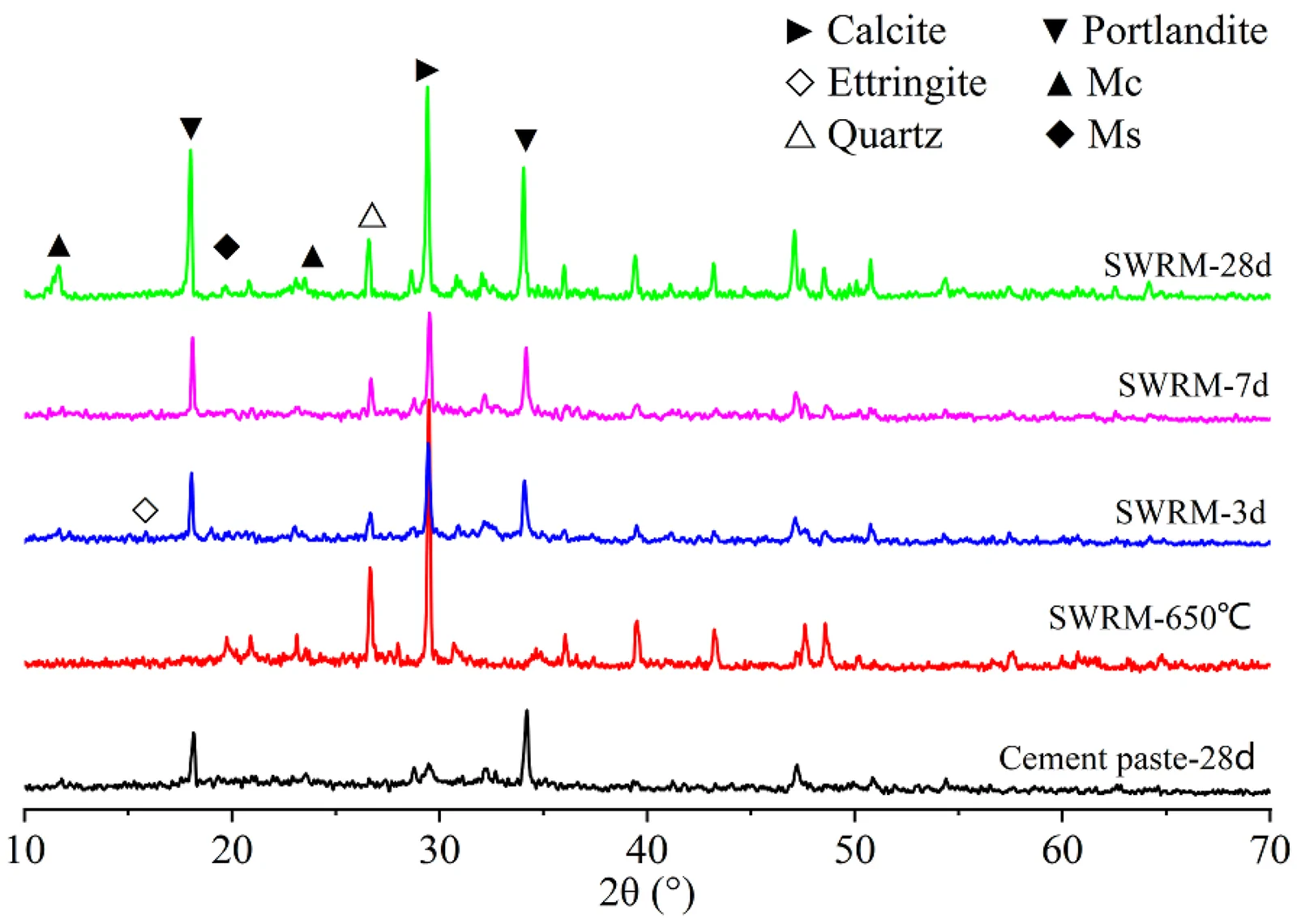
XRD of the mortar mixed with 30% of the SWRM from 650 °C calcination.

Figure 9 shows the XRD patterns of the mortar with 30% of SWRM mixed and calcined at 650 °C at different ages. The hydration products are mainly Ca(OH)_2_, calcium aluminosilicate (AFt), C-(A)-S-H, and monocarbonated calcium aluminate (Mc), etc. XRD patterns of the 3 d and 7 d age mortar specimens are not significantly different. In the early stage of hydration, cement hydration produces a large amount of C-(A)-S-H and calcium aluminosilicate, which promotes the development of the early strength of the composite cementitious material. With the increase in age, the characteristic peaks of C-(A)-S-H gradually strengthen. This is because Ca(OH)_2_ reacts with active alumina and silica to form C-(A)-S-H, and the C-(A)-S-H interweaves with other hydrated product crystals to form a dense network structure. As the hydration reaction proceeds, the crystal size continues to increase, promoting the development of the later strength of the composite cementitious material. In the 7 d XRD pattern, weak carbon aluminosilicate and monocarbonated sulfur aluminosilicate (Ms) characteristic peaks appeared, while the calcium aluminosilicate characteristic peak gradually decreased. This indicates that the amount of calcium aluminosilicate decreases, and it transforms into monocarbonated sulfur aluminosilicate. The activated calcium carbonate in the SWRM also begins to participate in the reaction, and CO_3_^2-^ reacts with aluminosilicate to form monocarbonated aluminosilicate. At the 28 d age, the characteristic peaks of monocarbonated aluminosilicate and monocarbonated sulfur aluminosilicate are higher. In the later stage of the reaction, there is still a large amount of Ca(OH)_2_ and calcium carbonate in the system, indicating that a large amount of Ca(OH)_2_ does not participate in the reaction and the reaction degree is low. Therefore, the strength of the fresh slurry with 30% calcined SWRM increases slowly in the later stage.

## 4. Mix Proportion Optimization of ASWRM-Based LC3 Cement

### 4.1. Optimization of Mix Proportion for ASWRM-Based LC3 Cement

Mixture design and optimization of LC3 cement were carried out with ASWRM, AC composed of cement clinker and 5% DG, and LP as independent variables, while the 7 d and 28 d compressive strengths were taken as response values. The mix module in Design-Expert 13 software was adopted for proportioning optimization. In the preliminary single-factor pre-test study, it was found that when the AC was lower than 40%, the compressive strength of the mortar was less than 20 MPa, and when it was higher than 80%, it had a negative impact on the fluidity. Therefore, the AC content was selected to be between 40 and 80%. When the proportion of ASWRM was 15%, the compressive strength of the mortar could reach 37.7 MPa. Based on the premise of increasing the application amount of ASWRM, the proportion of ASWRM used was selected to be between 15 and 40%. The LP content was 10%. The 28 d strength of the mortar reached its maximum value of 30.2 MPa. Considering the amounts of AC and ASWRM, the LP content was selected to be between 5 and 20%. The total mass fraction of all components was fixed at 100%. The experimental factors and levels are listed in Table 5, and the mix proportions of the 16 groups together with their compressive strength test data are summarized in Table 6.

### 4.2. Analysis of Influencing Factors

The special cubic model embedded in Design-Expert software was adopted to fit the 7 d and 28 d compressive strength, and the established multivariate nonlinear regression equations are shown in Equations (1) and (2). The analysis of variance results for the regression models is presented in Table 6.

(1) $$F_{C,7d}=28.34A-1.63B-16.39C+27.26AB+51.07AC+66.46BC-110.69ABC$$

(2) $$F_{C,28d}=37.01A+0.45B-23.27C+32.14AB+69.92AC+85.14BC-108.65ABC$$

In response surface methodology, the fitting quality is judged according to the *p*-value. Generally speaking, *p* < 0.001 indicates an excellent correlation between fitted values and experimental data; *p* < 0.05 means the factor is statistically significant; and *p* > 0.05 represents an insignificant factor [26,27,28,29]. At a confidence level of α = 0.05, the significance of interaction effects between variables can be evaluated by the F-value and *p*-value. A higher F-value accompanied by a lower *p*-value corresponds to a more significant influence [30,31].

As listed in Table 7, the F-values of the 7 d and 28 d compressive strength models reach 146.14 and 441.05 respectively, with *p* < 0.0001, which demonstrates that Equations (1) and (2) are highly significant. For Equation (1), the correlation coefficient *R*^2^ = 0.9898 and adjusted determination coefficient *R*^2^_Adj_ = 0.9831. For Equation (2), *R*^2^ = 0.9966 and *R*^2^_Adj_ = 0.9944. These values verify the high reliability of the two regression equations, proving that ASWRM, AC, and LP dominate the compressive strength development. The lack-of-fit *p*-values of both models are greater than 0.05, revealing minor experimental errors. The coefficient of variation (*C.V.*) of the two equations is less than 10, which reflects small data dispersion and further confirms good model reliability. The signal to noise (S/N) ratio refers to the ratio of interpretable information to unexplainable random noise [32]. An *S/N* value above 4 is regarded as a reliable model.

In conclusion, Equations (1) and (2) can be reliably used to predict the 7 d and 28 d compressive strength of SWRM-based LC3 cement.

**Figure 10:**
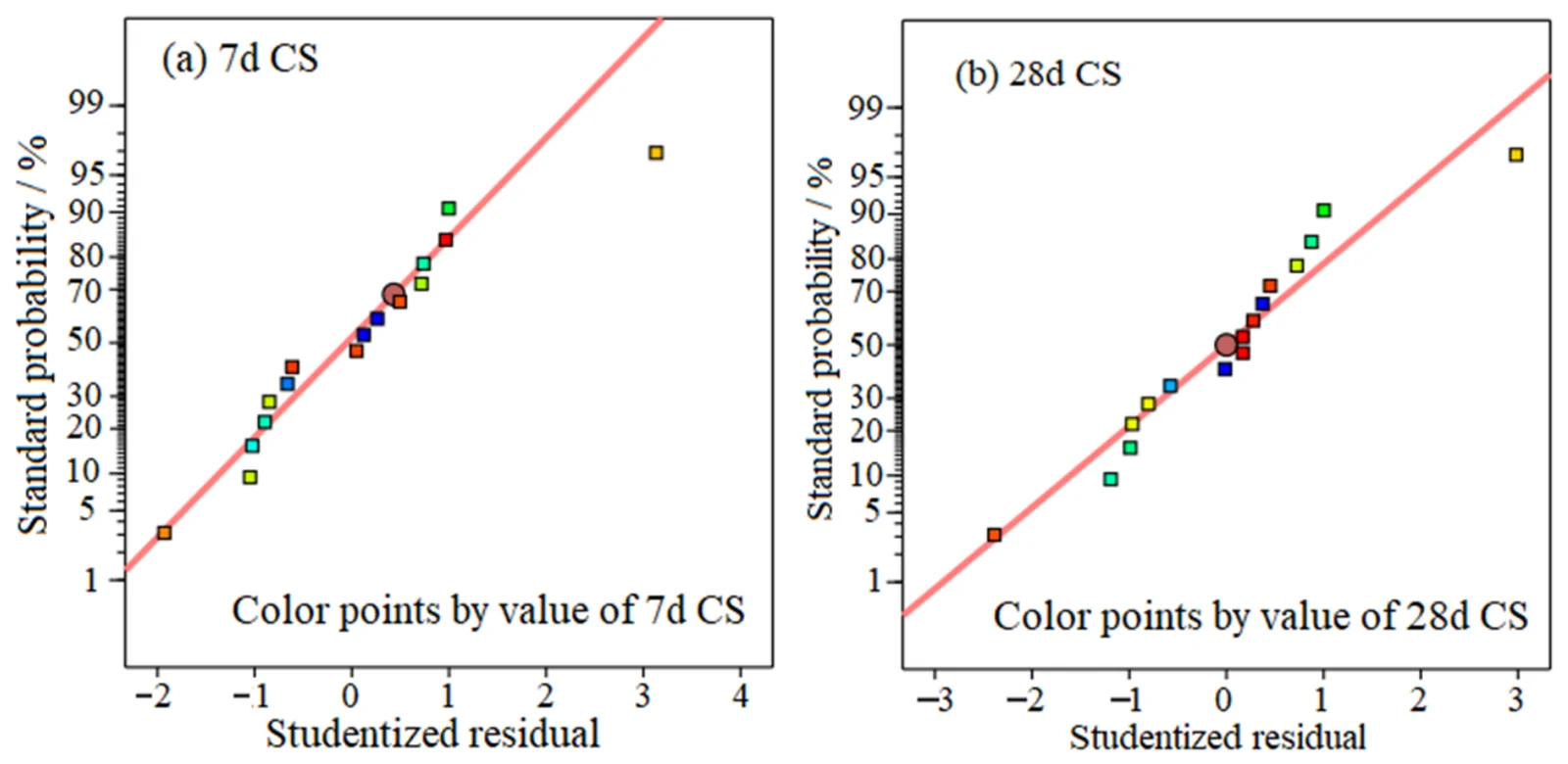
Residual analysis of 7 d and 28 d compressive strength.

Figure 10 shows the residual analysis for the 7 d and 28 d compressive strength. The residuals of the 7 d and 28 d compressive strength approximately follow a normal distribution, which means the deviations between predicted and measured values satisfy the normality assumption and further prove the good fitting performance of the regression model.

**Figure 11:**
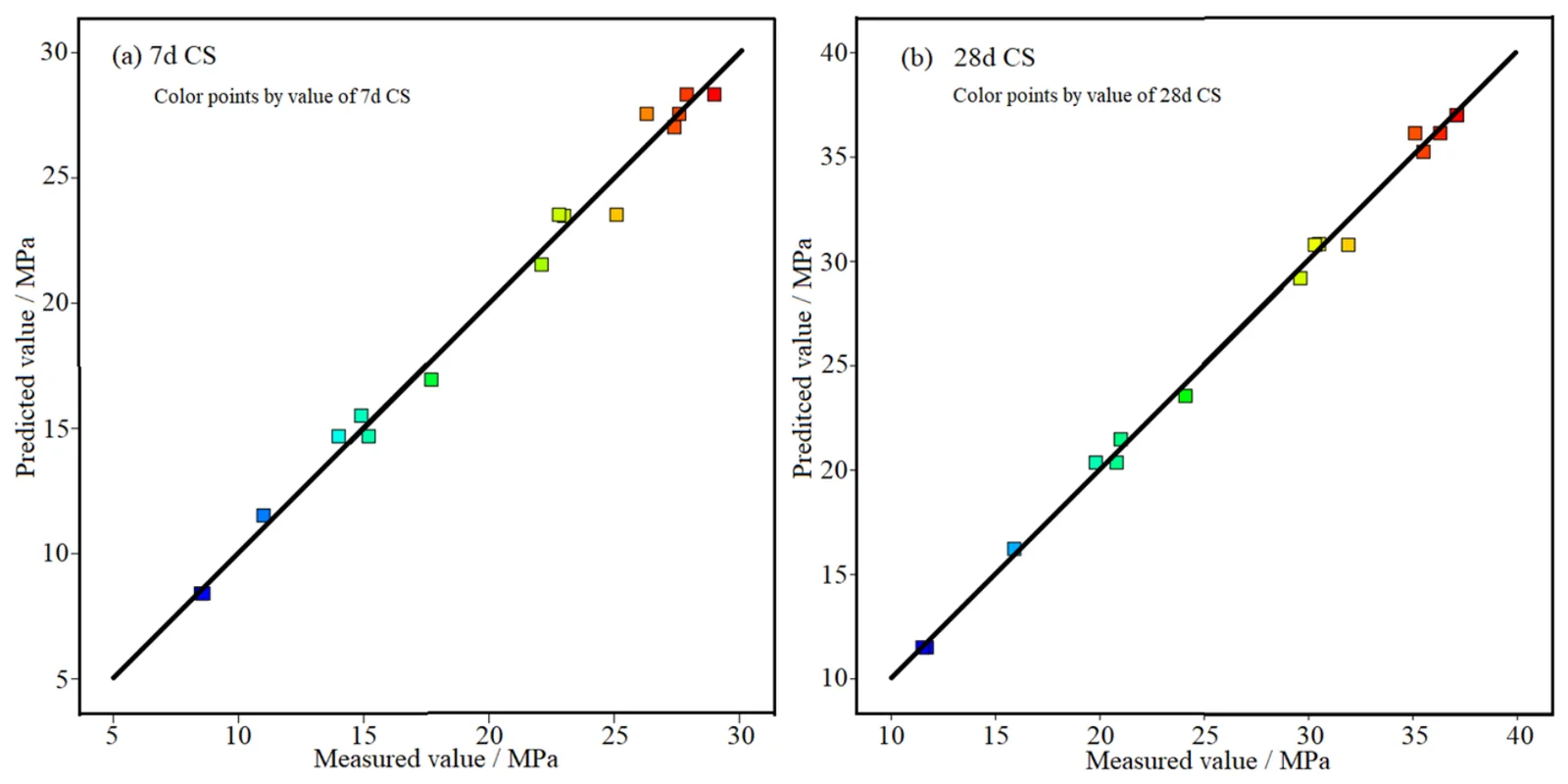
Comparison between measured and predicted values of 7 d and 28 d compressive strength.

Figure 11 is the scatter plot comparing the measured and predicted 7 d and 28 d compressive strength. It can be seen that all data points are evenly distributed around the diagonal line of y = x, indicating high consistency between model predictions and experimental measurements [33].

In summary, the regression models Equations (1) and (2) possess excellent fitting quality and high prediction reliability and can effectively predict the compressive strength of SWRM-based LC3 cement under practical experimental conditions.

To clearly reveal how the dosage of each component affects the compressive strength of SWRM-based LC3 cement, response surface plots and contour plots were established based on the regression analysis results, as shown in Figure 8. These figures visually illustrate the functional relationship and variation trend among the dosages of AC, ASWRM, and LP.

**Figure 12:**
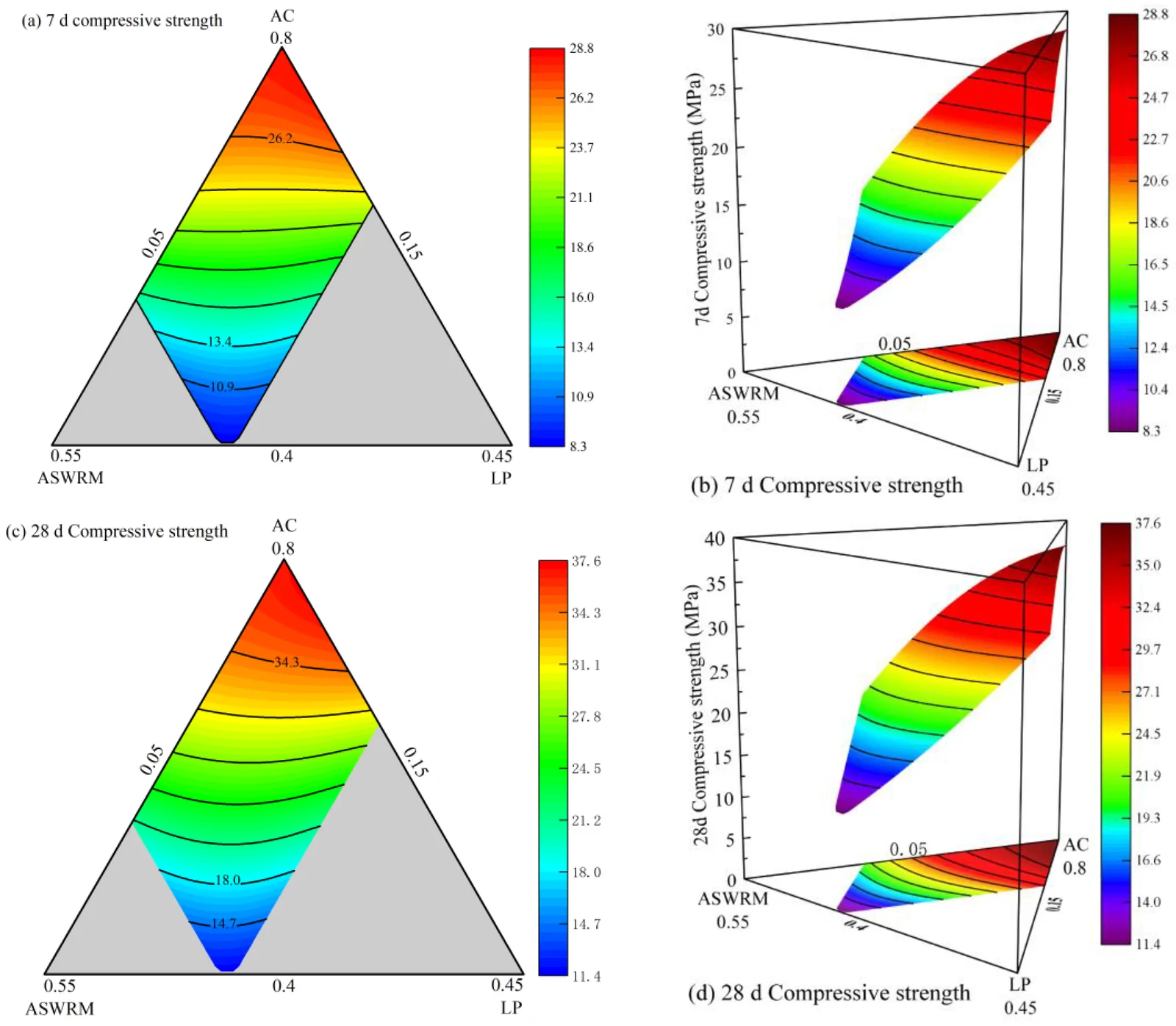
Influence of variable dosage on the compressive strength of specimen: (**a**,**b**) 7 d; (**c**,**d**) 28 d.

It can be seen from Figure 12 that the dosages of AC, ASWRM, and LP exert obvious nonlinear effects on the 7 d and 28 d compressive strength of LC3 cement. The 14th group in Table 5 achieves a compressive strength of 37.1 MPa, which indicates that the compressive strength of ASWRM-based LC3 cement mainly originates from the hydration reaction of AC cement. For Groups 2, 4, 8, and 11 in Table 5, when the content of ASWRM is fixed at 15%, the 28 d compressive strength of the cementitious material is no less than 30 MPa. Once the dosage of ASWRM is further increased, the 28 d compressive strength drops below 30 MPa. This proves that ASWRM possesses relatively low hydration activity. Excessive incorporation of ASWRM weakens the overall hydration degree of the binder system and consequently deteriorates the mechanical properties.

### 4.3. Determination of Optimal Mix Proportion of SWRM-Based LC3 Cement

**Figure 13:**
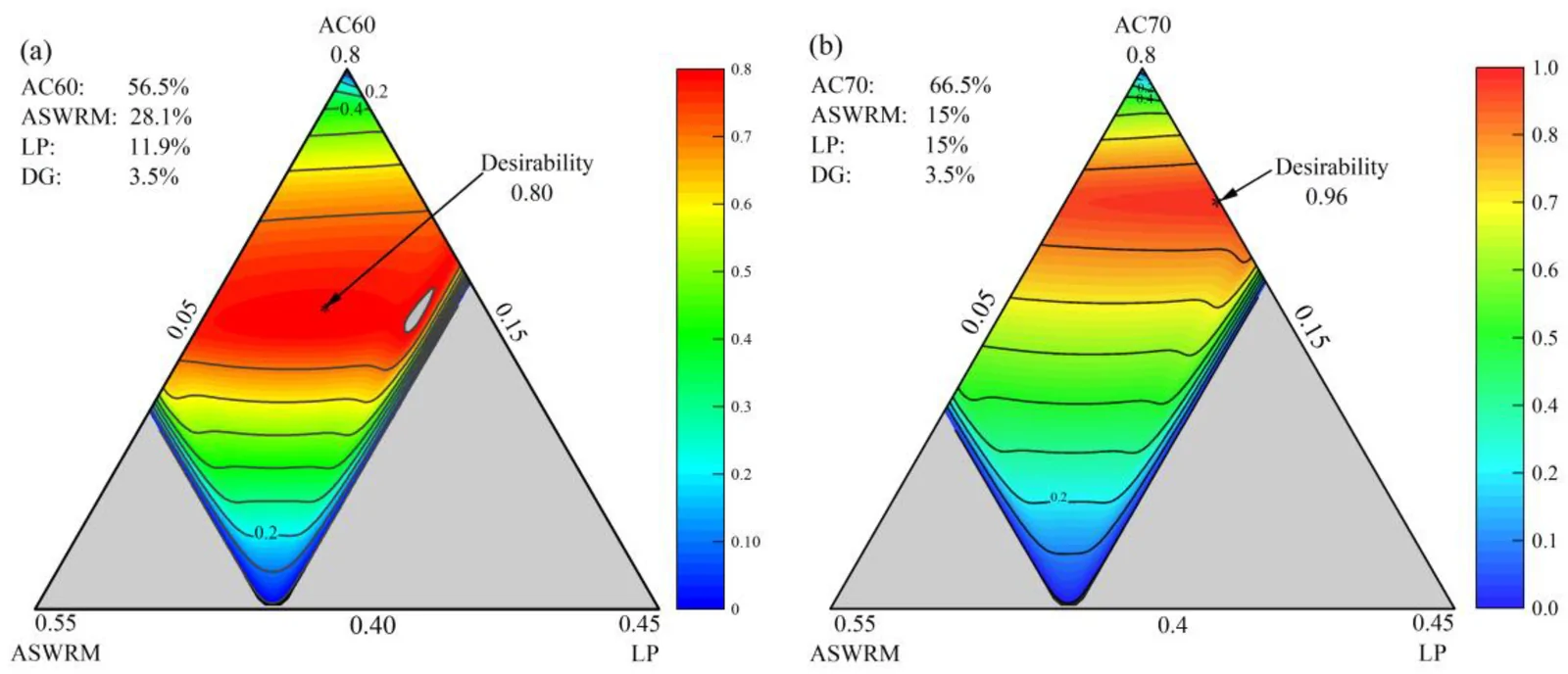
Desirability response surface results: (**a**) AC60; (**b**) AC70.

On the basis of the established strength fitting models, the optimization module in the mixture design was used to optimize the mix proportion. Considering the demand for cement strength in the project, the mix ratio optimization design of the SWRM-based LC3 cement was carried out with the aim of achieving the maximum 28 d compressive strength, as well as keeping the 7 d compressive strength and fluidity indicators within the specified range. To reduce the carbon emission of LC3 cement, the dosage of AC was reduced preferentially while making full use of ASWRM. Considering the mechanical strength requirements, the target dosages of AC were set to 60% and 70%. The corresponding desirability response surfaces obtained with these parameter settings are shown in Figure 13. A desirability value closer to 1 indicates a more reliable proportioning scheme [34,35].

Table 8 lists the optimal mix proportions calculated by the model when the target AC content was 70% and 60%, respectively. When the AC dosage was fixed at 70%, the desirability value for the 28 d compressive strength reached 0.96. When the AC dosage was set to 60%, the desirability value decreased to 0.80, revealing that AC content exerted a remarkable influence on strength development. According to the comparison between predicted and experimental values in Table 8, the relative errors of 7 d and 28 d compressive strength for both formulations are less than 5%. The models exhibit high fitting accuracy, favorable prediction performance, as well as excellent reliability and applicability.

According to the optimal mix proportion of SWRM-based LC3 cement shown in Table 8, mortar specimens were prepared for experimental verification, with P·O 42.5 cement set as the control group. To ensure the comparability of experimental results, the fluidity of all mortars was controlled within the range of 200–210 mm. The detailed mix proportions and fluidity data are presented in Table 9.

Table 10 presents the test results of water requirement of standard consistency, setting time and soundness for the three groups. It can be observed that the water requirement gradually rises in the sequence of the P group, AC70 group, and AC60 group with the increasing content of ASWRM. The value increases from 25.5% for the P group to 29.2% for the AC70 group and further to 30.8% for the AC60 group. This can be attributed to partial particle agglomeration inside ASWRM after high temperature calcination, which greatly enhances its water absorption capacity. The P group has the shortest initial and final setting times, which are 135 min and 175 min, respectively. The initial and final setting times of the AC70 group are 170 min and 240 min. Compared with the P group, the initial setting time is prolonged by 35 min, and the final setting time is prolonged by 65 min. Higher dosages of ASWRM and LP lead to longer initial and final setting times of LC3 cement. The incorporation of ASWRM and LP reduces the amount of early hydration products. Moreover, the dissolution and diffusion of ASWRM and LP depend on the alkali content in the system, which slows the depolymerization–condensation reaction of active components. Consequently, the formation of the structural network is delayed, resulting in longer setting times [36,37].

The experimental results demonstrate that the initial and final setting times of SWRM-based LC3 cement in the AC70 group and AC60 group meet the requirements of national standards, and all specimens pass the soundness test.

### 4.4. Mechanical Properties of SWRM-Based LC3 Cement

**Figure 14:**
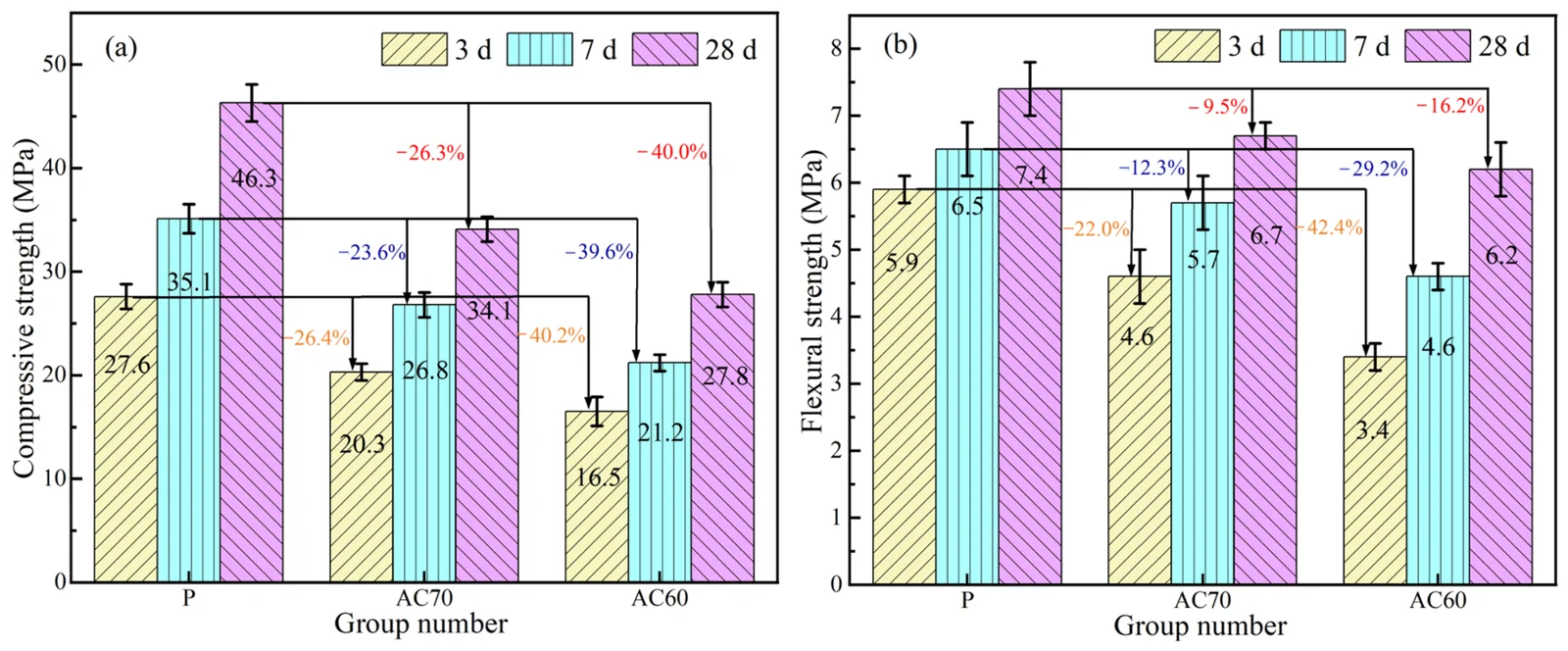
Mechanical properties: (**a**) compressive strength; (**b**) flexural strength.

Figure 14 compares the mortar strength of the P group, AC70 group and AC60 group. As shown in Figure 14a, the 28 d compressive strength of the P group reaches 46.3 MPa, while those of the AC70 group and AC60 group are 34.1 MPa and 27.8 MPa, respectively. In comparison with the P group, the 28 d compressive strength of the AC70 group and AC60 group decreases by 26.3% and 40.0%, which indicates that higher replacement ratios of ASWRM and LP lead to a more significant strength reduction of SWRM-based LC3 cement. It can be seen from Figure 14b that the 28 d flexural strength of the P group is 7.4 MPa, and the values for the AC70 group and AC60 group are 6.7 MPa and 6.2 MPa, respectively. The 28 d flexural strength declines by 9.5% and 16.2% correspondingly, following the same trend as compressive strength. The 3 d and 28 d strength of AC70 cement satisfies the technical specifications for Grade 32.5 cement. Considering practical engineering application, excessive addition of ASWRM impairs mechanical performance owing to its low content of active components. Combined with the experimental data, it is recommended that the total dosage of ASWRM and LP should not exceed 30%.

### 4.5. XRD

**Figure 15:**
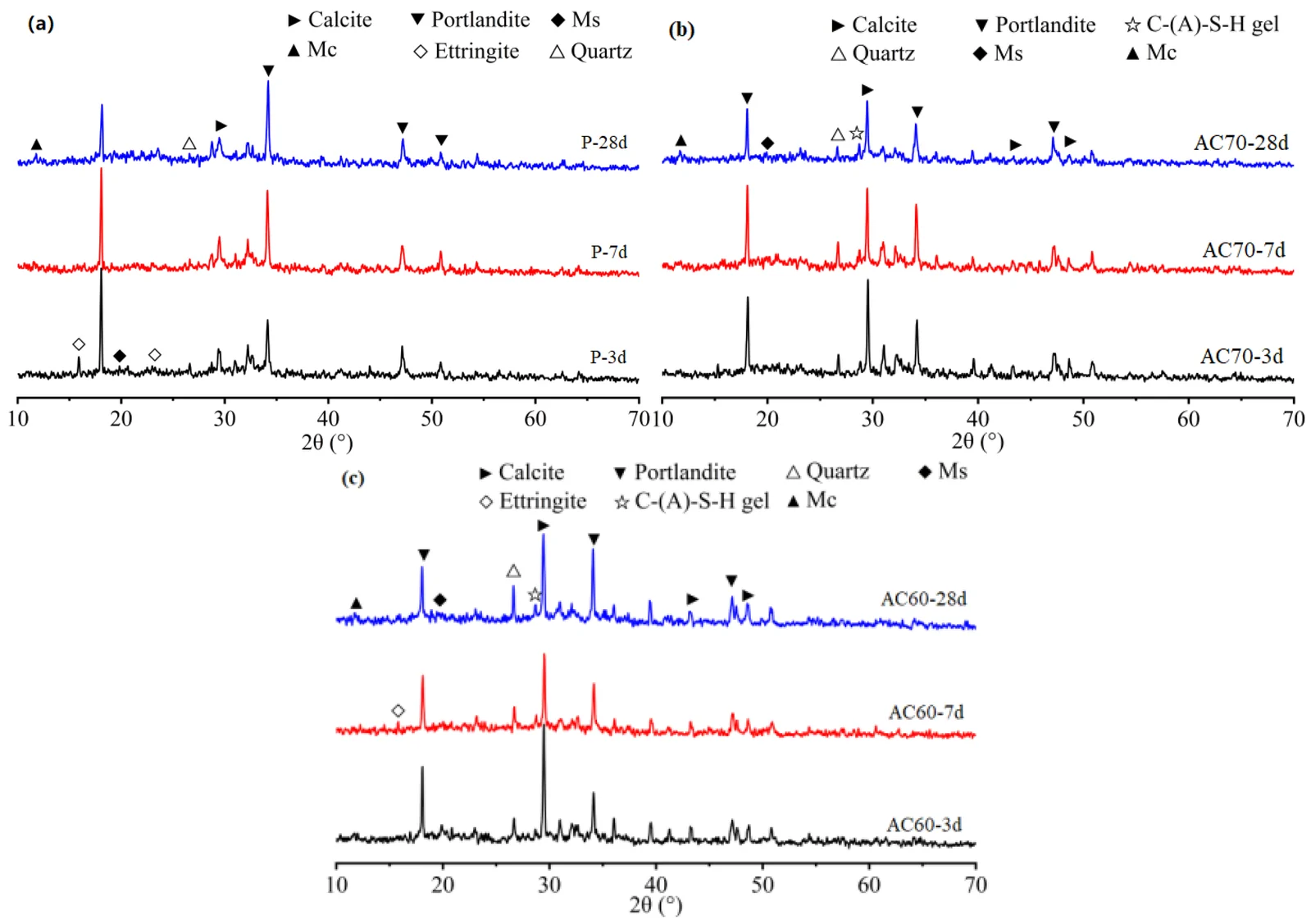
XRD patterns of the P group: (**a**) 3 d; (**b**) 7 d; (**c**) 28 d.

Figure 15 presents the XRD results of the P group, AC70 group, and AC60 group at different curing ages. As illustrated in Figure 15a, the main hydration products of the P group are AFt, calcium hydroxide (CH), and C-(A)-S-H, which exhibit strong characteristic diffraction peaks in XRD patterns. Tricalcium aluminate contains aluminum phases whose dissolution rate is lower than that of sulfate ions, so ettringite (AFt) is preferentially formed in the system [38]. With the extension of curing age, the characteristic peaks of C-(A)-S-H at 25–40° gradually intensify. As hydration proceeds, the production of calcium hydroxide (CH) exceeds its consumption, leading to stronger diffraction peaks and higher crystallinity of Ca(OH)_2_ [39]. In the later hydration stage, part of AFt transforms into monosulfate (Ms), accompanied by weakened ettringite peaks. Meanwhile, characteristic peaks of monocarboaluminate (Mc) can be observed at 28 d [40]. This phenomenon occurs because sulfate ions are exhausted before complete hydration. At a low carbonate concentration, the excess aluminum phases first react with carbonate to form hemicarboaluminate (Hc), which is further converted into Mc as the reaction continues [41]. The AFt generated in the early hydration stage makes a major contribution to early strength. It interweaves with C-(A)-S-H and other hydration products to form a dense network structure. The matrix becomes more compact, and the mechanical strength increases continuously during subsequent hydration.

Figure 15b,c show the XRD patterns of hydration products of SWRM-based LC3 cement in the AC70 and AC60 groups at different curing ages. As shown in Figure 11b, the main hydration products of SWRM-based LC3 cement include AFt, CH, and C-(A)-S-H. New diffraction peaks corresponding to Ms and Mc appear in the 7 d XRD pattern. The hydration products generated from cement clinker constitute the primary source of early strength for SWRM-based LC3 cement. As the hydration reaction proceeds, the diffraction peaks of C-(A)-S-H within the 20–40° become sharper and broader, and the content of CH increases continuously. This might be the result of the hydration process, where the cement clinker continuously undergoes hydration, generating more CH while also increasing the liquid phase alkalinity. More Al-O and Si-O bonds break, forming more C-(A)-S-H. At the same time, the amount of gypsum in the system decreases, the AFt phase transforms into the AFm phase, the crystal grains become smaller, and the XRD peaks broaden [42]; meanwhile, CaO in ASWRM hydrates to produce extra CH, which raises the alkalinity of the pore solution [36]. Reactive silica and alumina in ASWRM then react with CH to form abundant C-(A)-S-H that fills the pores of the matrix and improves mechanical strength [43]. The pozzolanic reaction of ASWRM proceeds slowly, resulting in residual CH and calcium carbonate (CaCO_3_) inside the hardened paste. This leads to a moderate strength gain at later ages, which is the primary reason why its strength at all curing ages is lower than that of the P group.

### 4.6. TG-DTG Analysis

**Figure 16:**
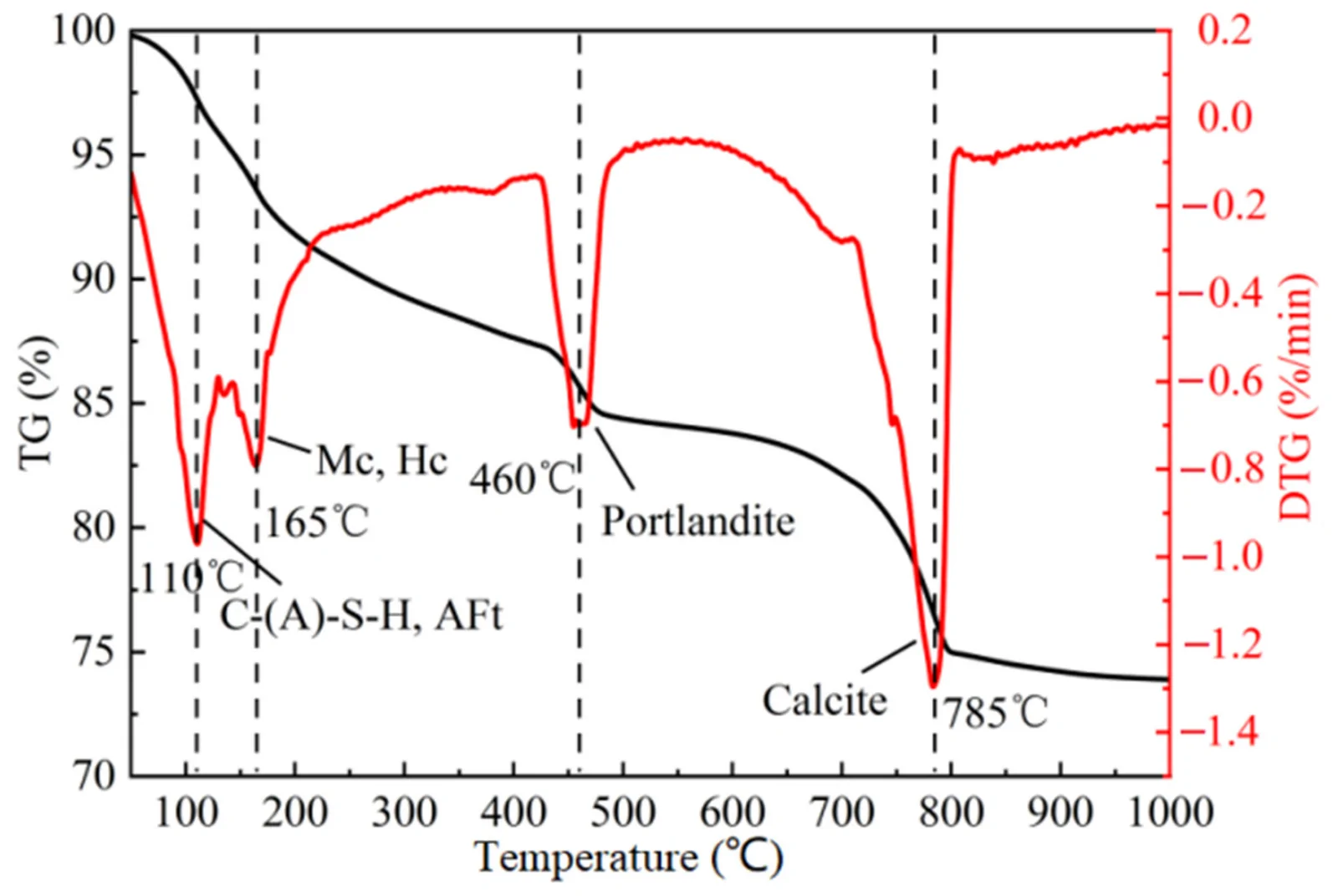
TG-DTG curves of the AC70 group at 28 d.

Figure 16 shows the TG-DTG curves of SWRM-based LC3 cement cured for 28 d. It can be seen from the curves that the hydration products experience four distinct mass loss stages. The mass loss in stage I between 90 °C and 122 °C is mainly caused by the decomposition of C-(A)-S-H and AFt [44]. Stage II occurs from 152 °C to 175 °C, which corresponds to the decomposition of Mc and Hc. It is difficult to distinguish the individual mass losses of these two minerals by TG-DTG [45]. Stage III ranging from 425 °C to 500 °C is attributed to the decomposition of Ca(OH)_2_ [46]. This indicates that a large amount of CH remains unreacted in the system, which accounts for the slow strength development at later ages. Stage IV between 710 °C and 807 °C corresponds to the thermal decomposition of CaCO_3_ [47,48].

Based on the weight loss values ranging from 425 °C to 807 °C obtained in the TG test, the content of Ca(OH)_2_ was calculated according to Equation (3). In the TG graph, the weight loss above 600 °C was mainly caused by the decomposition of calcium carbonate. Therefore, the content of its chemical bound water was calculated according to Equation (4) based on the weight loss values from 80 °C to 600 °C. The results show that the calcium hydroxide content is 21.8% and the chemical binding water content is 23.6%.

(3) $$M_{CH}=\frac{\omega_{425}-\omega_{500}}{\omega_{500}}\times \frac{74}{18}\times \frac{1}{m_{c}}$$

(4) $$M_{w}=(\omega_{80}-\omega_{600})\times \frac{1}{m_{c}}$$

w_t_ represents the percentage of weight loss at temperature t degrees Celsius, and m_c_ represents the mass percentage of cement clinker in this system.

### 4.7. FT-IR

**Figure 17:**
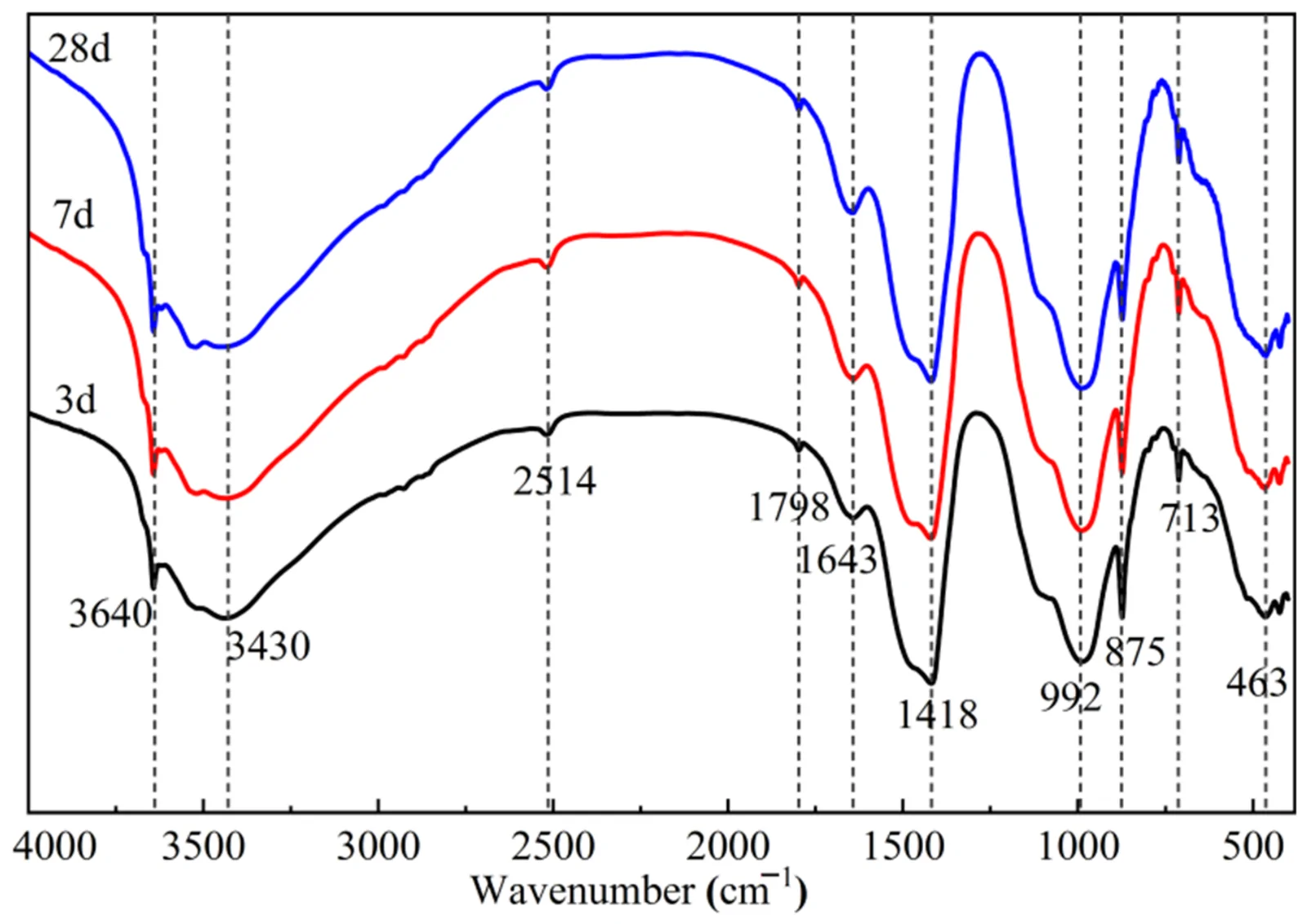
FT-IR spectrum of ASWRM-based LC3 cement.

Figure 17 shows the FT-IR spectra of the AC70 group recorded in the wavenumber range of 400 cm^−1^ to 4000 cm^−1^. The absorption band at 643 cm^−1^ is assigned to hydroxyl bending vibrations originating from CH [49], free water and hydroxyl groups in ASWRM, which proves that water participates in hydration and forms crystalline hydrates. A strong absorption peak near 992 cm^−1^ corresponds to bending vibrations and asymmetric stretching vibrations of Si-O bonds in C-(A)-S-H [50]. The stretching vibration band ν (Si-O) of Q^2^ at 992 cm^−1^ represents isolated tetrahedra linked by two bridging oxygen atoms to form long chains. As hydration proceeds continuously, this peak becomes broader and more intense, demonstrating that cement clinker continuously produces C-(A)-S-H, whose content rises with curing age. Meanwhile, the peak position slightly shifts toward higher wavenumbers. This phenomenon confirms that reactive silica from ASWRM takes part in the pozzolanic reaction, raising the Si/Al ratio in the matrix. Si-O bonds polymerize into Si-O-Si linkages, which stabilize the silicate tetrahedral structure [51]. The intergrowth of C-(A)-S-H and other hydration products builds a dense microstructure and effectively improves mechanical strength.

### 4.8. SEM Analysis

**Figure 18:**
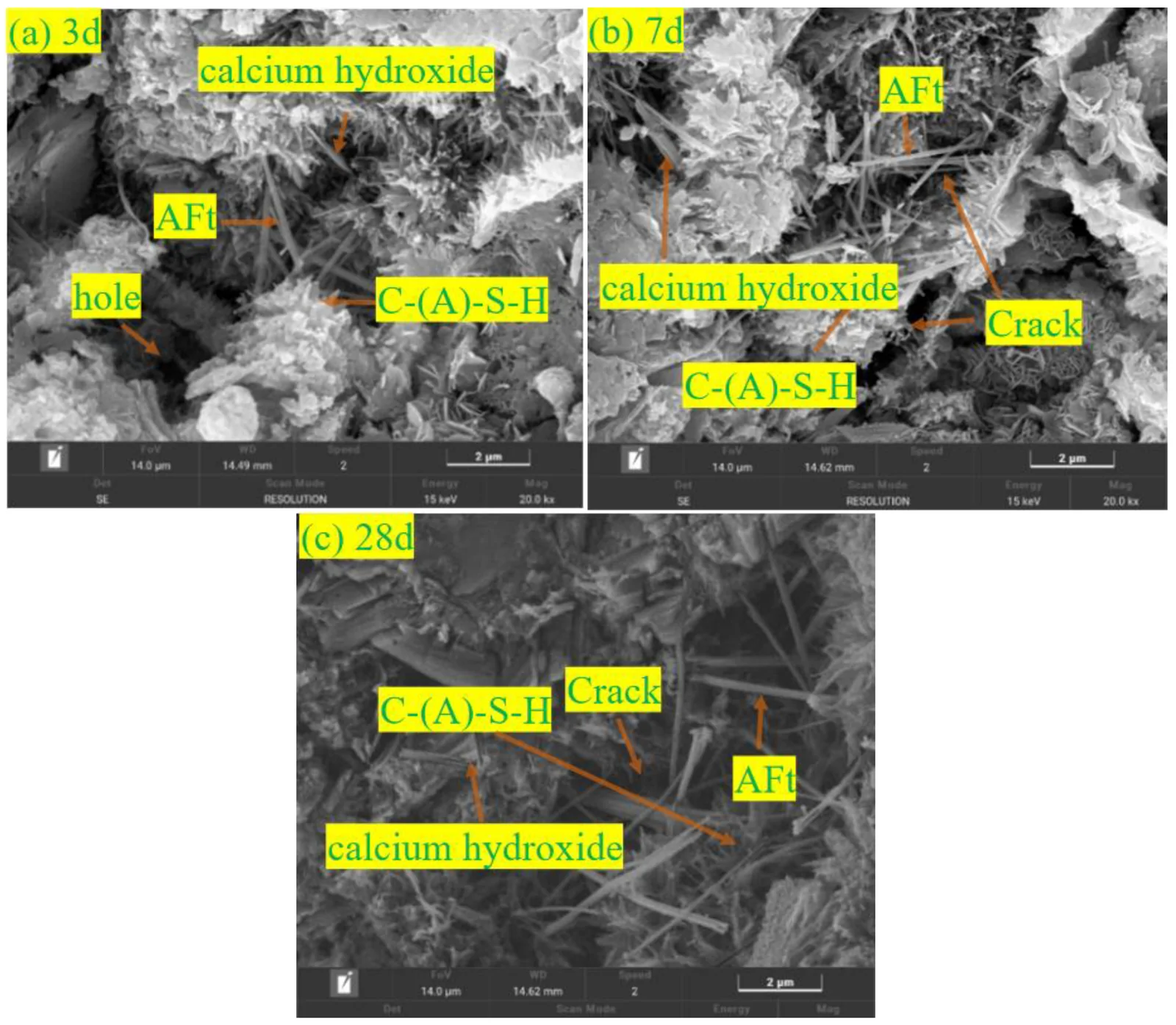
SEM test results: (**a**) 3 d; (**b**) 7 d; (**c**) 28 d.

Figure 18 shows the micro-morphology of hydration products of hardened paste specimens in the AC70 group cured for 3 d, 7 d, and 28 d.

As shown in Figure 18a, the main hydration products at 3 d are AFt, CH, and C-(A)-S-H, which are primarily derived from cement clinker. The partially hydrated ASWRM and LP are enwrapped by hydration products. At this stage, ASWRM and LP mainly play a filling effect, and provide nucleation sites and space for the subsequent growth of C-(A)-S-H and other hydration products [52].

It can be seen from Figure 18b that a large amount of honeycomb-like C-(A)-S-H forms in the specimen at 7 d [53]. AFt crystals interpenetrate and overlap within the C-(A)-S-H, and CH can also be observed in the matrix. The amount of hydration products increases significantly compared with the sample at 3 d, and the SWRM is fully covered by hydration products.

As illustrated in Figure 18c, a dense honeycomb microstructure is formed with the extension of curing age. The silicate tetrahedra and four-coordinated aluminate tetrahedra on the surface of ASWRM break down and undergo secondary hydration reactions with CH [54]. The hydration products distribute unevenly and interweave with those generated from cement clinker to form an interconnected network, making the matrix much denser than that at 7 d. Owing to the slow pozzolanic reaction of ASWRM, needle-like AFt and CH crystals can still be clearly observed, which is consistent with the XRD analysis results.

### 4.9. Ecological Benefits of the SWRM-Based LC3 Cement

The ecological benefit evaluation results of the SWRM-based LC3 cement and ordinary Portland cement are shown in Table 11. During the calculation, the EE (hidden energy of the cementitious material) of CC, ASWRM, LP, and DG are 5.5 MJ/kg, 0.9 MJ/kg, 0.85 MJ/kg, and 1.8 MJ/kg respectively; the EC (carbon emissions of the cementitious material) are 0.735 kgCO_2_ e/kg, 0.08 kgCO_2_ e/kg, 0.032 kgCO_2_ e/kg, and 0.12 kgCO_2_ e/kg respectively; and the costs of CC, ASWRM, LP, and DG are 0.4 yuan/kg, 0.06 yuan/kg, 0.22 yuan/kg, and 0.2 yuan/kg respectively. The selected material data have significant representativeness and are in line with the “Building Carbon Emission Calculation Standard” (GB/T 51366—2019) [55] and EN ISO14040 (2006) [56]. There may be small differences in energy consumption and carbon emissions due to different production processes and processing methods. The calcination temperature of SWRM is 650 °C, and it is converted according to the carbon emission of CC during the calcination process. The hidden energy, carbon emissions, and costs of raw materials are shown in Table 11.

As shown in Table 11, the implicit energy, carbon emissions and cost of the SRWM-based LC3 cement are all lower than those of ordinary silicate cement. The carbon emissions of the SRWM-based LC3 cement account for 49.2% to 56.1% of those of ordinary silicate cement. The AC60 group has the lowest implicit energy, carbon emissions and cost, which are 3.52 MJ/kg, 0.446 kgCO_2_ e/kg and 0.276 yuan/kg respectively. Compared with P, the implicit energy of the AC70 and AC60 groups decreases by 27.6% and 36.0% respectively, the carbon emissions decrease by 44.1% and 51.1% respectively, and the cost decreases by 25.0% and 34.3% respectively. The influence of cement clinker on the carbon emissions and cost of the SRWM-based LC3 cement accounts for about 60% and 50% respectively. This is because the carbon emissions and costs of cement clinker are relatively high. Increasing the content of ASWRM and limestone powder can also significantly reduce the carbon emissions and costs. Compared with ordinary silicate cement, the carbon emissions and costs of the SRWM-based LC3 cement are lower. Therefore, in the actual production process, it can promote the consumption of SWRM, limestone powder and DG and other solid wastes and reduce the carbon emissions in the construction industry.

**Figure 19:**
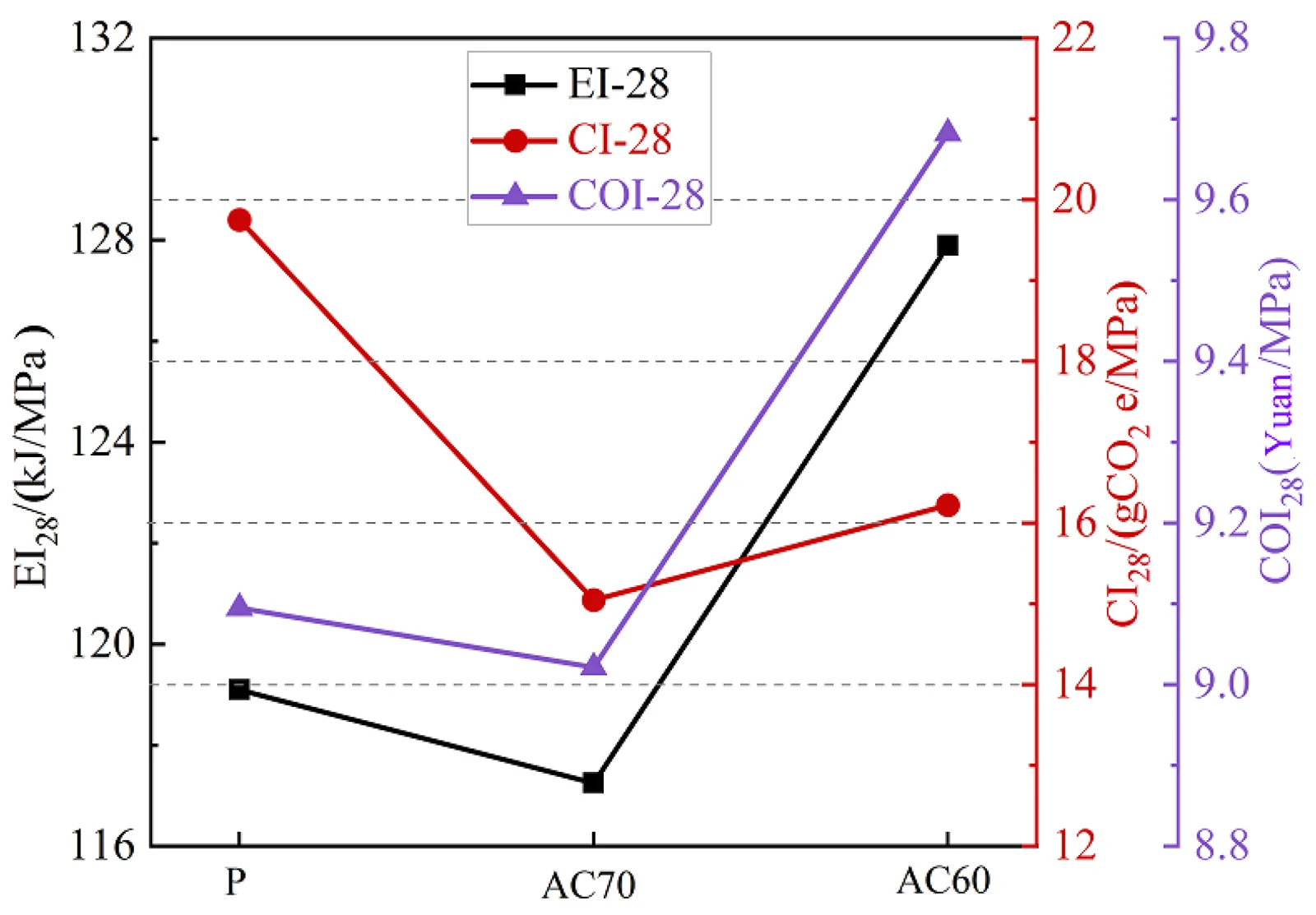
EI_i_, CI_i_, and COST_i_ of compressive strength for different groups of units.

In order to quantify the environmental impact and the material cost per unit compressive strength of the SWRM-based LC3 cement, the implicit energy index (*EI_i_*), carbon emission index (*CI_i_*), and material cost index (*COST_i_*) were calculated using Equations (5), (6), and (7) respectively. The implicit energy index, carbon emission index, and cost index per unit compressive strength for different groups are shown in Figure 19.

(5) $$EI_{i}=\frac{EE}{C_{i}}$$

(6) $$CI_{i}=\frac{EC}{C_{i}}$$

(7) $$COST_{i}=\frac{COST}{C_{i}}$$

In the Equation, *EI_i_* represents the implicit energy index, measured in kJ/MPa; *EE* represents the implicit energy of the cementitious material, measured in kJ·kg; *CI_i_* represents the carbon emission index, measured in gCO_2_ e/MPa; EC represents the carbon emission quantity of the cementitious material, measured in gCO_2_ e/kg; *COST_i_* represents the material cost index, measured in yuan/MPa; *COST* of the cementitious material represents the cost of the material, measured in yuan/MPa; and C_i_ represents the compressive strength.

From Figure 19, it can be seen that at the 28 d age, the EI_28_, CI_28_ and COST_28_ of the AC70 group’s SWRM-based LC3 cement were all lower than those of the ordinary Portland cement. Compared with the P group, the EI_28_, CI_28_ and COST_28_ of the AC70 group decreased by 1.6%, 23.8% and 0.1% respectively, while the EI_28_ and COST_28_ of the AC60 group increased by 7.4% and 6.5% respectively. Meanwhile, the CI_28_ of the AC60 group decreased by 17.9%. This indicates that when the ASWRM content is 15%, replacing a certain amount of cement clinker can significantly reduce the implicit energy, carbon emissions and cost per unit of compressive strength. Considering both environmental and economic impacts, when the ASWRM content is 15%, the SWRM-based LC3 cement has superior environmental and economic benefits.

## 5. Conclusions

In this study, SWRM was subjected to mechanical activation and thermal activation to produce a supplementary cementitious material with potential hydraulic activity. The main conclusions are summarized as follows:

(1) Within a ball milling duration of 9 min, the particle size of raw SWRM gradually decreases, and the specific surface area increases with prolonged milling. After 3 min of grinding, the specific surface area exceeds 1000 m^2^/kg and reaches 1220 m^2^/kg after 9 min of grinding, which is 1.4 times that of the raw material. For SWRM ground for more than 3 min, the particle size is mainly distributed in the range of 0.1–30 μm. When the milling time exceeds 9 min, further grinding leads to slight particle agglomeration, which results in a marginal increase in particle size and a slight reduction in specific surface area.
(2) For SWRM ground for 3 min, its pozzolanic activity first rises and then falls with the increase in calcination temperature. The optimal activation temperature is 650 °C, with the 3 d, 7 d, and 28 d activity indices reaching 69.5%, 84.7%, and 79.3%, respectively. When the calcination temperature exceeds 850 °C, particle sintering and agglomeration occur, leading to a decrease in the activity index.
(3) The relative error between the predicted values of the LC3 cement strength model based on SWRM established through the mixture optimization design module and the measured compressive strength of the AC70 group of SWRM-based LC3 cement after model optimization is less than 5%. This proves that this model can be used for the strength prediction and composition ratio optimization of SWRM-based LC3 cement.
(4) The optimization results from the response surface model reveal that when the dosage of AC cement is 70%, and the dosages of ASWRM and LP are both 15%, the prepared SWRM-based LC3 cement achieves a 28 d compressive strength of 34.1 MPa. The initial and final setting times are 170 min and 240 min, the water requirement of standard consistency is 29.2%, and the soundness meets the standard requirements.
(5) The characterizations including XRD, TG-DTG, FT-IR, and SEM images demonstrate that under the combined effect of CH and DG, the silicate and aluminate tetrahedra in ASWRM undergo hydrolysis to form C-(A)-S-H, which is the major contributor to the late-age strength development of SWRM-based LC3 cement.
(6) When the proportion of ASWRM is 15%, the WRSM-based LC3 cement has superior environmental and economic benefits. Compared with ordinary cement, the implicit energy of the SWRM-based LC3 cement is reduced by 27.6%, the carbon emissions are decreased by 44.1%, and the cost is reduced by 25%.

The above research is based on the results of a specific study on SWRM waste materials. The research mainly focuses on the activation mechanism and mechanical properties. As a promising new type of SWRM-based cementitious material, for its application in engineering, further research is needed to improve aspects such as volume deformation and environmental durability.

## Figures and Tables

**Table 1:** Chemical compositions of raw materials.

Materials	SiO_2_	CaO	Al_2_O_3_	Fe_2_O_3_	K_2_O	MgO	TiO_2_	SO_3_	Other
SWRM	36.88	32.24	14.32	6.83	5.66	2.77	0.79	0.21	0.3
CC	22.45	64.34	5.12	3.44	0.52	1.39	—	0.42	1.86
DG	2.24	38.97	1.49	0.56	0.4	0.84	—	54.92	0.58
Cement	24.52	61.34	5.26	3.64	0.31	1.32	—	2.68	0.93

**Table 2:** Performance index of cement.

Material	Density (g·cm^−3^)	Specific Surface Area (m^2^/kg)	Setting Time (min)		Compressive Strength (MPa)	
			Initial Set	Final Set	3 d	28 d
Cement	2.98	360	145	210	24.6	46.1

**Table 3:** Characteristic particle size of SWRM.

Characteristic Particle Size (μm)	Grinding Time (min)					
	0	3	6	9	12	15
D_10_	1.66	0.98	0.93	0.85	0.89	0.93
D_50_	9.89	3.50	3.06	2.58	2.76	3.01
D_90_	74.43	14.27	12.48	10.17	10.42	11.41

**Table 4:** The particle size distribution of SWRM at different calcination times.

Calcination Temperature	D_10_ (μm)	D_50_ (μm)	D_90_ (μm)	Specific Surface Area (m^2^/kg)
0 °C	0.98	3.50	14.27	1080
650 °C	1.01	3.77	16.58	980
750 °C	0.99	3.76	17.37	970
850 °C	1.26	5.72	34.41	720

**Table 5:** Factors and levels.

Factor	NO.	Level		
		−1	0	1
AC/%	A/%	40	60	80
ASSW/%	B/%	15	27.5	40
LP/%	C/%	5	12.5	20

**Table 6:** Experimental mix proportion and test results.

NO.	AC (%)	ASWRM (%)	LP (%)	Compressive Strength (MPa)	
				7 d	28 d
1	55	32	13	17.7	24.1
2	65	15	20	22.8	30.3
3	40	40	20	8.6	11.5
4	72	15	13	27.6	36.3
5	65	15	20	25.1	31.9
6	62	25	13	22.1	29.6
7	73	22	5	27.4	35.5
8	72	15	13	26.3	35.1
9	47	33	20	11.0	15.9
10	54	40	7	14.0	20.8
11	80	15	5	29.0	37.1
12	54	26	20	14.9	21.0
13	53	40	7	15.2	19.8
14	80	15	5	27.9	37.1
15	65	30	5	23.0	30.5
16	40	40	20	8.5	11.7

**Table 7:** Analysis of variance table for regression model.

Source	Compressive Strength							
	Sum of Squares		d_f_		F-Value		*p*-Value	
	7 d	28 d	7 d	28 d	7 d	28 d	7 d	28 d
Model	768.47	1209.50	6	6	146.14	441.05	<0.0001	<0.0001
Linear Mixture	749.99	1178.85	2	2	47.87	1289.61	<0.0001	<0.0001
AB	9.33	12.97	1	1	10.64	28.37	0.0098	0.0005
AC	7.17	13.44	1	1	8.18	29.40	0.0188	0.0004
BC	7.14	11.73	1	1	8.15	25.65	0.0189	0.0007
ABC	11.58	11.10	1	1	13.21	24.41	0.0054	0.0008
Residual	7.89	4.11	9	9	—	—	—	—
Lack of fit	3.07	1.59	4	4	0.7956	0.7904	0.5757	0.5872
Pure error	4.82	2.52	5	5	—	—	—	—
Cor total	776.35	1213.62	15	15	—	—	—	—
	7 d				28 d			
	*R*^2^ = 0.9898 $R_{Adj}^{2}$* *= 0.9831 *C.V.* = 4.66 *S/N* = 32.17				*R*^2^ = 0.9966 $R_{Adj}^{2}$* *= 0.9944 *C.V.* = 2.53 *S/N* = 57.03			

**Table 8:** Comparison table between predicted and measured compressive strength.

NO.	Factor (%)				Compressive Strength (MPa)				Error (%)	
					Predicted Value		Measured Value			
	CC	ASWRM	LP	DG	7 d	28 d	7 d	28 d	7 d	28 d
AC60	56.5	28.1	11.9	3.5	21.4	28.4	21.2	27.8	0.9	2.1
AC70	66.5	15.0	15.0	3.5	26.5	34.8	26.8	34.1	1.1	2.0

**Table 9:** Mix ratios of cement materials.

NO.	P·O 42.5	CC	ASWRM	LP	DG	w/b	Water Reducer (g)	Fluidity (mm)
P	100%	—	—	—	—	0.5	0	205
AC70	—	66.5%	15%	15%	3.5%	0.5	0.61	210
AC60	—	56.5%	28.1%	11.9%	3.5%	0.5	0.79	200

**Table 10:** Setting time, standard consistency, and stability of different systems.

NO.	Initial Setting Time (min)	Final Setting Time (min)	Water Requirement of Normal Consistency
P	135	175	25.5%
AC70	170	240	29.2%
AC60	190	280	30.8%

**Table 11:** Embodied energy, embodied carbon, and cost of materials.

Materials	EE/(MJ/kg)	EC/(kgCO_2_ e/kg)	Cost/(Yuan/kg)
CC	5.5	0.735	0.40
ASWRM	0.90	0.080	0.06
LP	0.85	0.032	0.22
DG	1.80	0.120	0.20
AC70	3.98	0.510	0.315
AC60	3.52	0.446	0.276
P	5.50	0.912	0.420

## Data Availability

The original contributions presented in this study are included in the article. Further inquiries can be directed to the corresponding authors.

## Abbreviations

The abbreviations in the manuscript are as follows: sand washing residue mud is abbreviated as SWRM, activated sand washing residue mud is abbreviated as ASWRM, cement clinker is abbreviated as CC, limestone powder is abbreviated as LP, desulfurization gypsum is abbreviated as DG, and the cement clinker containing 5% desulfurized gypsum is abbreviated as AC.
